# Necrosis and ethylene‐inducing‐like peptide patterns from crop pathogens induce differential responses within seven brassicaceous species

**DOI:** 10.1111/ppa.13615

**Published:** 2022-08-05

**Authors:** Henk‐jan Schoonbeek, Hicret Asli Yalcin, Rachel Burns, Rachel Emma Taylor, Adam Casey, Sam Holt, Guido Van den Ackerveken, Rachel Wells, Christopher J. Ridout

**Affiliations:** ^1^ Department of Crop Genetics John Innes Centre Norwich UK; ^2^ Plant–Microbe Interactions, Department of Biology Utrecht University Utrecht Netherlands; ^3^ Present address: Department of Metabolic Biology John Innes Centre NR4 7UH Norwich UK; ^4^ Present address: The Scientific and Technical Research Council of Turkey (TÜBITAK), Marmara Research Centre Genetic Engineering and Biotechnology Institute Kocaeli Turkey; ^5^ Present address: Centre of Plant Sciences, Faculty of Biological Sciences University of Leeds LS2 9JT Leeds UK; ^6^ Pacific Biosciences Ltd. Rolling Stock Yard 188 York Way London N7 9AS UK

**Keywords:** brassica, grey mould, grey mould, microbe/pathogen‐associated molecular pattern, necrosis and ethylene‐inducing peptide 1‐like proteins

## Abstract

Translational research is required to advance fundamental knowledge on plant immunity towards application in crop improvement. Recognition of microbe/pathogen‐associated molecular patterns (MAMPs/PAMPs) triggers a first layer of immunity in plants. The broadly occurring family of necrosis‐ and ethylene‐inducing peptide 1 (NEP1)‐like proteins (NLPs) contains immunogenic peptide patterns that are recognized by a number of plant species. *Arabidopsis* can recognize NLPs by the pattern recognition receptor AtRLP23 and its co‐receptors SOBIR1, BAK1, and BKK1, leading to induction of defence responses including the production of reactive oxygen species (ROS) and elevation of intracellular [Ca^2+^]. However, little is known about NLP perception in *Brassica* crop species. Within 12 diverse accessions for each of six *Brassica* crop species, we demonstrate variation in response to *Botrytis cinerea* NLP BcNEP2, with *Brassica oleracea* (CC genome) being nonresponsive and only two *Brassica napus* cultivars responding to BcNEP2. Peptides derived from four fungal pathogens of these crop species elicited responses similar to BcNEP2 in *B*. *napus* and *Arabidopsis*. Induction of ROS by NLP peptides was strongly reduced in *Atrlp23*, *Atsobir1* and *Atbak1‐5 Atbkk1‐1* mutants, confirming that recognition of *Brassica* pathogen NLPs occurs in a similar manner to that of HaNLP3 from *Hyaloperonospora arabidopsidis* in *Arabidopsis*. In silico analysis of the genomes of two *B*. *napus* accessions showed similar presence of homologues for *AtBAK1*, *AtBKK1* and *AtSOBIR1* but variation in the organization of *AtRLP23* homologues. We could not detect a strong correlation between the ability to respond to NLP peptides and resistance to *B*. *cinerea*.

## Graphical abstract

All *Brassica* species except *B. oleracea* recognize pathogen NLPs which, unlike *Arabidopsis*, was not correlated with disease control highlighting challenges of translational research from models to crops.

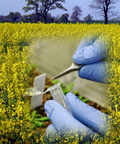

## 1 INTRODUCTION

The Brassicaceae is one of the largest families in the plant kingdom, with 338 genera and over 3700 species. Six species of major agricultural and horticultural importance in the genus *Brassica* are defined by their genome arrangement based on observations by (Nagaharu, 1935) in a relationship described as the "triangle of U". The genomes of diploid species *Brassica rapa* (AA), *B. nigra* (BB) and *B. oleracea* (CC) are hybridized in the allotetraploid (amphidiploid) species *B. juncea* (AABB), *B. napus* (AACC) and *B. carinata* (BBCC) (commonly referred to as polyploids). Oilseed rape or canola (*B*. *napus*) is the most important temperate oilseed crop in the world, with a production of close to 70 million tonnes and the third most important crop in the United Kingdom after wheat and barley. Other brassica species include oilseeds and vegetables such as broccoli, cabbage and cauliflower (*B*. *oleracea*), bok choi, field mustard and turnip (*B*. *rapa*), Ethiopian mustard and Abyssinian cabbage (*B*. *carinata*) and brown and black mustard (*B*. *juncea* and *B*. *nigra*, respectively). The Brassicaceae also includes *Arabidopsis thaliana*, facilitating translation of basic research to application in *Brassica* crops.

Pathogens cause diseases of *Brassica* crops that reduce yields and quality (Zheng et al., 2020). *B*. *napus* is affected by stem canker, or blackleg disease, caused by *Leptosphaeria maculans* and *L. biglobosa*, light leaf spot (*Pyrenopeziza brassicae*) and Verticillium stem striping (*Verticillium longisporum*). Other important fungal pathogens of brassicas include *Alternaria brassicicola*, *Sclerotinia sclerotiorum*, and the closely related *Botrytis cinerea*. Brassicas are affected by oomycetes including *Peronospora parasitica* (downy mildew) and *Albugo candida*, and bacterial pathogens including *Pseudomonas syringae* and *Xanthomonas* species.

High disease pressure and decreased availability or efficacy of crop protection chemicals puts increased emphasis on breeding for plant resistance against pathogens. While major *R* gene‐mediated resistance has been widely used in breeding, pathogens can overcome this, as illustrated in the evolution of virulence in *L*. *maculans* infecting *B*. *napus* (Neik et al., 2017). One approach to address this problem is breeding for quantitative disease resistance (QDR), which pathogens are less likely to overcome, to provide more durable disease control. QDR genes are difficult to identify in crop species, but translational research based on insight from *Arabidopsis* can accelerate their discovery. For example, *AtCYP81F2*, the orthologue of the suspected candidate gene for BLMR2, a QDR locus against *L*. *maculans* in *B*. *napus*, encodes a cytochrome P450 enzyme involved in defence in *Arabidopsis* (Zhang et al., 2021). Pattern recognition receptor‐triggered immunity (PTI), extensively studied in *Arabidopsis* (Boutrot & Zipfel, 2017), is potentially one mechanism contributing to QDR.

Pathogen‐ or microbe‐associated molecular patterns (PAMPs/MAMPs) are molecular motifs present in diverse taxonomic phyla that activate innate immunity. Well‐studied MAMPs include chitin, flagellin and elongation factor Tu (EF‐Tu). Short amino acid peptides of flagellin (flg22) can induce PTI in many plant species whereas EF‐Tu (elf18) induces PTI in *Arabidopsis* and other Brassicaceae. Detection by pattern recognition receptors (PRRs), which include receptor‐like kinases (RLKs) and receptor‐like proteins (RLPs) (Boutrot & Zipfel, 2017), activates quantitative or qualitative defence responses including the production of reactive oxygen species (ROS) and gene induction. We previously described methods to evaluate PTI responses in *B*. *napus* (Lloyd et al., 2014, 2017).

Necrosis‐ and ethylene‐inducing proteins (NEPs) and NEP‐like proteins (NLPs), secreted by oomycetes, fungi and bacteria, can be cytotoxic or noncytotoxic depending on the host (Seidl & Van den Ackerveken, 2019) and can contribute to virulence. Whereas BcNEP1 and BcNEP2 are not required for virulence of *B*. *cinerea* on tomato (Arenas et al., 2010), silencing by application of double‐stranded (ds)RNA shows a weak contribution of BcNEP2 to virulence of *B*. *cinerea* on *B*. *napus* and a strong effect of SsNEP2 from *S*. *sclerotiorum* (McLoughlin et al., 2018). LmNLP1 from *L*. *maculans* induces necrosis and contributes to virulence on *B*. *napus* 'Topas' (Haddadi et al., 2016). Apart from these studies, little is known about the recognition or contribution to virulence of NLPs from *Brassica* pathogens.

NLPs can be classified into types with different effects on plant response (Oome & Van den Ackerveken, 2014). The type 1 subfamily contains a 20–24 amino acid fragment sufficient for eliciting PTI, which is defined by the microbial species name from which it is derived, for example BcNEP2 from *B. cinerea*. Type 2 NLPs can also elicit cytotoxicity but lack the motif that induces PTI (Oome et al., 2014). The 24 amino acid type 1 motif in NLPs is a MAMP‐like elicitor recognized by a receptor‐like protein (RLP)‐type PRR (Albert et al., 2015; Bohm et al., 2014) and activates PTI. Oome and Van den Ackerveken (2014) showed that the amino acid sequence of the MAMP motif of type 1 NLPs from *Brassica* pathogens is highly conserved (Table 1). The 20 amino acid MAMP motif PpNLP20 from *Phytophthora parasitica* induced ethylene production, a signature of PTI, in several Brassicaceae species (Bohm et al., 2014).

**TABLE 1:** Sequence and source of NLP‐PAMP motifs and corresponding peptides used in this study

Protein	Length^a^	Amino acid sequence	Organism	Host	Role in virulence	Expressed in planta^b^	Reference
PccNLP	12/26	GSFYALYFLKDQ.ILNGVNSGHRHDWE	*Pectobacterium carotovorum*	Potato, wide	Partial [1]	ND [1]	ZP_03832719
BsNPP1	18/24	AIMYSWYFPKD…EPSPGLGHRHDWE	*Bacillus subtilis*	Soil	ND		WP_003220392.1
HaNLP3	17/24	AIMYAWYFPKD…SPMLLMGHRHDWE	*Hyaloperonospora arabidopsidis*	*Arabidopsis*	ND	Yes [2]	JQ027036.1
AbNLP2	16/24	AIMYSWYMPKD…SPGPGLGHTHDWE	*Alternaria brassicicola*	Brassicas	ND	Yes	AB00887
BcNEP1	16/27	GIMYAWYFPKDQPAAGNVVGGHRHDWE	*Botrytis cinerea*	Broad, including Brassicaceae	No [3]	Yes [3]	NC_037311.1
BcNEP2	17/24	AIMYSWYMPKD…EPSTGIGHRHDWE	*B. cinerea*	Broad, including Brassicaceae	Partial [3, 4]	Yes [3, 4]	XM_001550999.2
LmNLP1	17/24	AIMYSWYMPKD…SPGPGLGHRHDWE	*Leptosphaeria maculans*	Brassicas, mainly *Brassica napus*	Yes [5]	Yes [6]	CBX91493.1
VlNLP	15/24	AILYAWYMPKD…APSSGLGHRHDWE	*Verticillium longisporum*	Mainly *B. napus*	ND	ND	BN1723_004663
PAMP motif	18/24	AIMYsWYfPKd…xpxxgxGHRHDWE^c^					[7, 8]

^a^ Number of amino acids matching the consensus motif/total length of peptide to cover the motif area.

^b^ References describing protein or expression, if available: [1] Mattinen et al. (2004), [2] Cabral et al. (2012), [3] Arenas et al. (2010), [4] McLoughlin et al. (2018), [5] Haddadi et al. (2016), [6] Sonah et al. (2016), [7] Bohm et al. (2014), [8] Oome & Van den Ackerveken (2014). ND, not determined.

^c^ Amino acids in upper case are highly conserved, those in lower case moderately; each dot indicates an amino acid position in alignment gaps.

In *Arabidopsis*, the leucine‐rich receptor (LRR)‐like protein RLP23 mediates immune activation by NLPs from oomycetes (*P. parasitica*, PpNLP; *Hyaloperonospora arabidopsidis*, HaNLP3), fungi (*B*. *cinerea*, BcNEP2) or bacteria (*Bacillus subtilis*, BsNPP1) (Albert et al., 2015; Ono et al., 2020). Out of 135 *Arabidopsis* accessions tested, only three lacked recognition of NLPs due to the same mutation in RLP23 (Albert et al., 2015). RLP23 forms a complex with the LRR receptor kinase (LRR‐RK) SOBIR1 (Suppressor of Brassinosteroid insensitive 1 [BRI1]‐associated kinase [BAK1]‐interacting receptor kinase 1) and recruits a second LRR‐RK, BAK1, triggering the cascade of PTI defence reactions. The contribution of somatic embryogenesis receptor kinases (SERKs) to PpNLP20 responses has been shown in the *Arabidopsis* double mutant *bak1‐5 bkk1‐1* affected in both SERK3/BAK1‐ and SERK4/BAK1‐LIKE1(BKK1)‐mediated signalling (Albert et al., 2015). Various combinations of SERKs are co‐receptors for PTI in *Arabidopsis* (Roux et al., 2011). The *B*. *napus* resistance gene *LepR3* encodes an RLP and requires functional SERKs and NbSOBIR for operational expression in *Nicotiana benthamiana* plants, suggesting homologues of these genes contribute to resistance in *B*. *napus* (Ma & Borhan, 2015) as well as *Arabidopsis* (Albert et al., 2015).

Translational research is necessary to advance fundamental discoveries on model plants towards practical application in agriculture. *B*. *cinerea* is an extensively studied model pathogen with a multifaceted infection strategy that infects *Arabidopsis* and also *Brassica* crop species and so is an ideal pathogen for our studies. Because RLP23‐mediated recognition of NLPs contributes some resistance against *B*. *cinerea* in *Arabidopsis* (Ono et al., 2020), we considered that this could potentially provide a novel mechanism of QDR in *Brassica* crop species. Using BcNEP2 as a representative NLP known to induce immune responses, we examined recognition of this peptide in 12 accessions per representative *Brassica* species that cover diverse crop types and origins (Table S1). We tested whether peptide patterns found in NLPs from other *Brassica* pathogens might be recognized differently both in brassicas and in *Arabidopsis* mutants with defects in NLP recognition. Translational research must also take account of the different genomic context between model plants and crop species, many of which are polyploids. We therefore undertook a preliminary analysis in *B*. *napus* of the genome landscape of candidate *RLP23* and co‐receptor gene homologues that have been implicated in NLP perception in *Arabidopsis*.

## 2 MATERIALS AND METHODS

### 2.1 Plant material and growth

*Brassica* accessions comprised a range of cultivars, breeders' lines, landraces and uncultivated accessions. Twelve accessions from each *Brassica* species were obtained from seed in personal collections or the Germplasm Resource Unit at the John Innes Centre unless stated otherwise (Table S1). *B*. *oleracea* ‘Dwarf Blue’ and *B*. *rapa* ‘ChifuCNS’ and ‘Ryou’ were provided by Sujit Tha Shrestha (CN Seeds Ltd). Plants of the additional *B*. *oleracea* diversity set are from the Warwick GRU/UK Vegetable Genebank (UKVGB) and BBSRC sLoLa Brassica Rapeseed and Vegetable Optimisation (BRAVO) via Steven Penfield (JIC, UK). *Arabidopsis* Columbia, the overexpression line 35S:GcaMP3 and the mutants *rlp23‐1*, *rlp23‐2*, *sobir1‐12*, *sobir1‐13*, *bak1‐5* and *bak1‐5 bkk1‐1* were described previously (Albert et al., 2015; Roux et al., 2011; Vincent et al., 2017). Plants were grown in Levington F2 with 15% 4 mm grit in growth chambers for 5–7 weeks at 20–22°C/18–20°C day/night under TL‐tubes with a near‐sunlight spectrum at about 100 μmol·m^−2^·s^−1^ with a photoperiod of 10 h.

### 2.2 Pathogen growth conditions

*B*. *cinerea* strains B05.10 and ΔBcatrB4, a camalexin‐sensitive B05.10‐derived gene replacement mutant with reduced virulence on plants producing indole alkaloid defence compounds, were grown as described previously (Stefanato et al., 2009) and used for inoculations as described (Lloyd et al., 2014) with minor modifications. Spore suspensions (200 μl at 2.5 × 10^6^ spores/ml) were spread on 1/10 potato dextrose agar (PDA; 2.5 g/L potato dextrose broth, 12 g/L agar), grown for 24 h at 21°C, and 4 mm agar plugs were used for brassica inoculations of 22 mm leaf discs on 0.6% water agar. Lesion diameter was measured after 48 h at 21°C with a relative humidity of 85%–100% and low light (10–20 μmol⋅m^−^^2^⋅s^−^^1^). *Arabidopsis* wild‐type and mutant plants were droplet‐inoculated with *B*. *cinerea* at 2.5 × 10^5^ spores/ml in 5 μl of 6 g/L potato dextrose broth, and lesion diameter was measured after 72–96 h.

### 2.3 MAMP preparations

Sequences for NLPs were used as previously (Oome & Van den Ackerveken, 2014) or retrieved from public databases by BLAST searches using HaNLP3 and BcNEP2 as query and aligned using ClustalW (http://www.clustal.org/). The 24–27 amino acid sequence covering motifs I and II (Table 1) was ordered at 87.5% purity from GenScript and dissolved in dimethyl sulphoxide (DMSO) at 10 mM prior to dilution to stock solutions in sterile water. Flg22 (QRLSTGSRINSAKDDAAGLQIA) was ordered from Peptron and resuspended in sterile water at 10 mM. Crab shell chitin (NA‐COS‐Y) was provided by Yaizu Suisankagaku Industry Co. and suspended in sterile water (100 g/L) and autoclaved at 121°C for 15 min. All MAMP stock solutions were aliquoted and stored at −20°C.

### 2.4 Measurement of ROS

ROS measurements were performed with a luminol/peroxidase‐based assay (Lloyd et al., 2017). For each accession, two leaf discs (4 mm diameter) were cut with a Miltex biopsy puncher (Integra) from the second most recently expanded leaf of four individual plants and incubated in 200 μl sterile water in a 96‐well plate for 16–24 h in the dark. The water was replaced by a solution containing 34 mg/L luminol, 20 mg/L horseradish peroxidase (HRP), and the MAMP to be tested. Luminescence was recorded every 30 s over a 40 min period using a Varioskan Flash plate reader (Fisher Scientific) and displayed as the sum of photon counts. A minimum of two replicate assays were performed. The measurements were transformed to smoothed curves using the average of five adjacent measurements in a sliding window over all 80 time points. This was used to calculate the time point to the maximum response (top of peak; *T*_max_), the time point where the response started to unequivocally increase (defined as 1/3 of the maximum measurement; *T*_start_) and the time from start to maximum (*T*_up_ = *T*_max_ − *T*_start_) (Figure S1). The six brassica diversity panels were screened using 100 nM flg22 and BcNEP2 at a concentration range from 0.4 to 1250 nM on selected *B*. *napus* and *Arabidopsis* accessions.

### 2.5 Measurement of intracellular [Ca^2+^]

Intracellular Ca^2+^ concentrations [Ca^2+^]_cyt_ were measured in *Arabidopsis* Col‐0 plants expressing the green fluorescent protein (GFP)‐based Ca^2+^ sensor 35S:GCaMP3 by quantification of GFP fluorescence, on a Varioskan Flash luminometer (excitation 485 nm, emission 520 nm, 160 ms per well). Leaf discs were sampled as for ROS assays and measured for 10 min prior to challenge with MAMP solutions in water. The lowest fluorescence in this baseline period was designated F_0_ and determined for each well. Upon replacement of water with water or MAMP solution, fluorescence was measured every 30 s for 40 min, with *F*_t_ indicating fluorescence at that particular time point. Relative changes in fluorescence intensity reflecting the change in [Ca^2+^]_cyt_ were calculated as Δ*F* = (*F*_t_ − *F*_0_)/*F*_0_. A water treatment was included on the same plate as negative control. Total Δ*F* was calculated by adding up all 80 measurements in a 40 min period, corresponding to the area under the curve as depicted in Figure S8. The effect of flg22 and all NLPs on total Δ*F* was tested at 50 nM, and the effect of BcNEP2 on total and timing of [Ca^2+^]_cyt_ was measured at a concentration range from 2 to 250 nM.

### 2.6 Gene expression assays

Gene expression studies were performed as described (Lloyd et al., 2017; Schoonbeek et al., 2015) with slight modifications. After overnight incubation, water was replaced with fresh water, 2.5 × 10^5^ spores/ml *B*. *cinerea*, or one of the following PAMP solutions: 100 nM flg22, 100 nM BcNEP2 or 500 mg/L chitin. After 1 or 3 h, all liquid was removed and the samples were flash‐frozen in liquid nitrogen, pulverized in a Genogrinder (SPEX SamplePrep) at 1000 SPM (strokes per min) for 2 × 60 s and RNA extracted using the RNeasy plant kit (QIAGEN). Genomic DNA was removed using DNase Turbo (Ambion) and 1 μg RNA used for first‐strand cDNA synthesis in 20 μl with Superscript IV reverse transcriptase (Invitrogen). For quantitative PCR (qPCR), 0.2 μl cDNA was used in 14 μl with SYBR Green mastermix (Sigma Aldrich) in 384‐well plates on a Lightcycler 480 (Roche Life Science) with three biological and three technical repeats per sample. The number of cycles (*C*_t_) to go over the machine default threshold was averaged for technical repeats, and wells with abnormal melting curves were omitted. The amount of each test gene present in a sample was calculated as 2^−*C*t(test gene)^ and normalized as relative amount for each sample by dividing that number by the amount of normalizing gene in that sample (2^−*C*t[normalizing gene]^). For induction values, we divided the normalized amount of test gene by the normalized amount of the control sample treated with water at the same time point.

Expression of *B*. *napus* genes was normalized to *BnEf1α* (Lloyd et al., 2017) and *B*. *cinerea* genes to *BcEf1α* (Stefanato et al., 2009). qPCR primers for *Bcnep1* and *Bcnep2* were designed on sequences from cloned genes (Arenas et al., 2010). To obtain more discriminatory MAMP‐inducible marker genes, we tested candidate genes from the RNAseq experiment described previously (Lloyd et al., 2014) with the following criteria: basal level at least 0.1% of *BnEf1α* and induction at least fourfold at 1, 3 h or both time points in more than half of the homeologues. Primers were designed using Primer3Plus (http://www.bioinformatics.nl/primer3plus) to yield fragments between 80 and 160 bp (Table S3).

### 2.7 In silico identification of *B*. *napus* genes homologous to *Arabidopsis* genes involved in NLP recognition

The cDNA sequences of AtRLP23 (*At2g32680*.*1*), AtSOBIR1(*At2g31880*.*1*), AtSERK3/BAK1 (*At4g33430*.*1*), and AtSERK4/BKK1 (*At4g33430*.*1*) were used to identify homologues in the genomes of Zhongshuang 11 (http://cbi.hzau.edu.cn/bnapus/) (Song et al., 2020) and Darmor‐*bzh* v. 4.1 (http://www.genoscope.cns.fr/brassicanapus) (Chalhoub et al., 2014).

The tomato sequences used as outgroup in the construction of phylogenetic trees were obtained by BLAST searches of the *Arabidopsis* proteins against the public databases and selecting the corresponding genomic regions of annotated genes *SlSERK3B* (NM_001246942) for *AtBAK1* and *AtBKK1*, *SlSOBIR1* (NM_001315496) for *AtSOBIR*, and *SlCf‐9* (XM_019211290.2) as representative RLP with homology to AtRLP23.

The SequenceServer (http://sequenceserver.com) graphical user interface was used to create a database with each *B*. *napus* genome and the threshold for BLASTN analyses was set to over 60% identity with a minimum of 90% coverage for *AtRLP23* and *AtSOBIR1* orthologues and 70% for *AtBAK1* and *AtBKK1* orthologues. The sequences were retrieved by SAMtools software (Li et al., 2009). Reciprocal BLAST of retrieved *B*. *napus* genomic sequences was performed against *A. thaliana* (taxid:3702) with BLASTN to ensure the homologues were correctly assigned to gene families and IDs were assigned to each orthologue according to their location on the chromosome (Table S5). Karyotype images of the orthologues were created by R v. 3.6.1 with the ggbio package (https://www.bioconductor.org/packages/release/bioc/html/ggbio.html). Genomic sequences of the orthologues were aligned with Clustal Omega (https://www.ebi.ac.uk/Tools/msa/clustalo/). Conserved region across alignments were selected by trimming the untranslated regions (UTRs) with poor local alignments at the beginning and end of each sequence. After initial alignment, the UTR for all genes was taken out at start or stop codons, if present, or at their expected position if they were absent, and the trimmed sequences were realigned to visualize phylogeny. Neighbour‐joining trees (Saitou & Nei, 1987) were built with Clustal Omega with default settings and visualized with iTOL v. 5.5 (https://itol.embl.de/).

### 2.8 SNP calling and prediction of functional effects of variations

Whole genome sequencing data of Darmor (Tudor et al., 2020) were aligned to the Darmor‐*bzh* reference genome and SNP‐calling performed using freebayes (https://github.com/freebayes/freebayes) v. 1.1.0.46. bcftools software (https://samtools.github.io/bcftools/) v. 1.8 was used to exclude the variants with a read depth <20, 600 quality and 0.95 alignment index. Sequence variants located within the genes of interest were extracted using bcftools. To annotate and predict the variant effects, SnpEff toolbox was used at (http://snpeff.sourceforge.net/).

### 2.9 Statistics

All data from independent repeat experiments were compiled in Excel 365 (Microsoft) and used for statistical analysis in GenStat v. 14 (https://vsni.co.uk/software/genstat) with experiment set as blocking factor in analysis of variance (ANOVA).

## 3 RESULTS

### 3.1 Measurement of ROS within *Brassica* species

**FIGURE 1:**
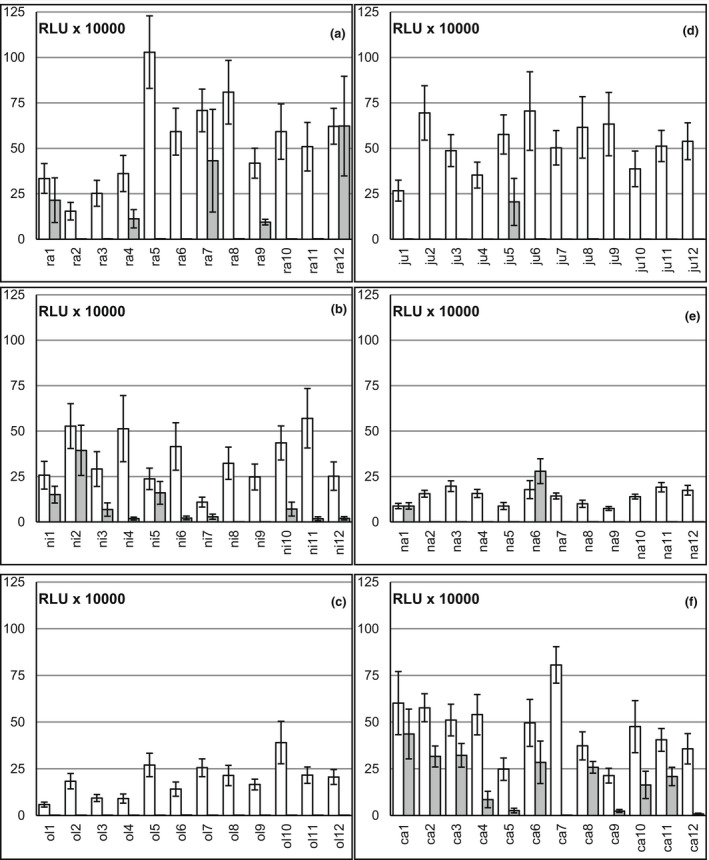
Some, but not all, *Brassica* accessions in the triangle of U recognize BcNEP2. For each species the reactive oxygen species (ROS) response to 100 nM flg22 (white) or 100 nM BcNEP2 (grey) was measured in 12 accessions. (a) *B*. *rapa* (AA), (b) *B*. *nigra* (BB), (c) *B*. *oleracea* (CC), (d) *B*. *juncea* (AABB), (e) *B*. *napus* (AACC), (f) *B*. *carinata* (BBCC). Bars represent means of at least eight different plants per accession (±*SEM*), measured as total accumulated relative luminescence units (RLUs) over 40 min.

**FIGURE 2:**
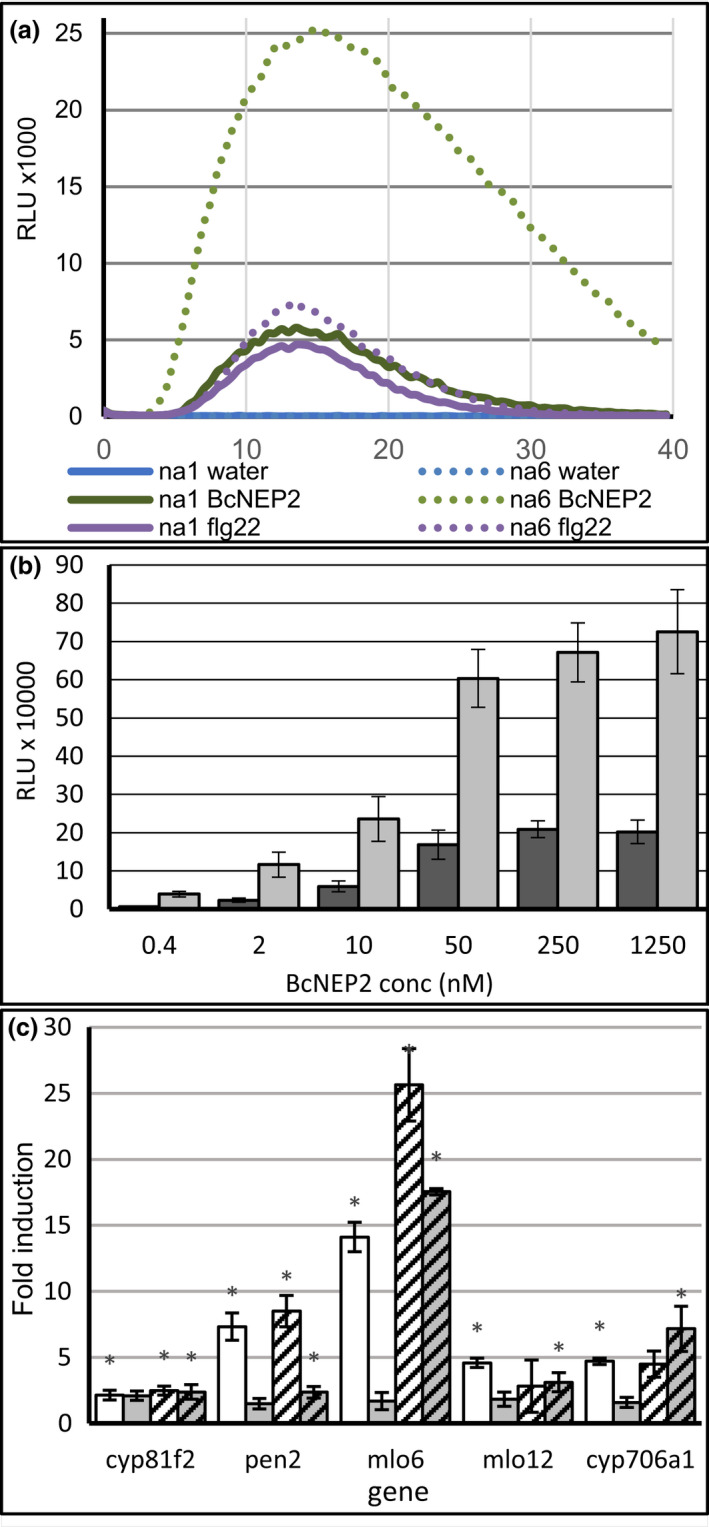
Recognition of MAMPs in *Brassica napus*. (a) The reactive oxygen species (ROS) response in two cultivars na1 (Ningyou1; solid line) and na6 (N02D; dotted line) after treatment with water (blue), 50 nM flg22 (purple) or 50 nM BcNEP2 (green) for 40 min with relative luminescence units (RLUs) measured every 30 s. Curves represent the mean of eight leaf discs in one representative experiment, which was repeated three times with similar results. (b) Induction of ROS in response to BcNEP2 is concentration dependent in na1 (Ningyou1; dark grey) and na6 (N02D; light grey). Bars display the mean (±*SEM*) of eight leaf discs in one representative experiment, which was repeated three times with similar results. (c) MAMP‐induced gene expression in *B*. *napus* na6 (N02D) relative to the water‐treated control. Preinfiltrated leaf discs were treated with 100 nM flg22 for 1 h (white) or 3 h (grey) or with 100 nM BcNEP2 for 1 h (white, striped) or 3 h (grey, striped). Bars represent the mean (±*SEM*) induction of three individual treatments relative to the water controls. Treatments were compared by analysis of variance and significant differences (*p* < 0.05) between treatment and water are indicated with * [Colour figure can be viewed at wileyonlinelibrary.com]

We tested 12 accessions of each *Brassica* species for ROS production after 100 nM flg22 or 100 nM BcNEP2 peptide treatment. All accessions could respond to flg22 (Figure 1) with transient ROS production (Figure 2a and Figure S1, Table 2). The highest proportion of BcNEP2‐responding accessions was in *B*. *carinata* whereas not a single *B*. *oleracea* accession responded (Figure 1). To exclude the possibility that nonresponsiveness in *B*. *oleracea* was due to biased selection of accessions, we tested a further 79 lines and did not find a single plant responding to BcNEP2 (Table S2a). In an additional set of 10 *B*. *rapa* accessions we found two other responding accessions (Table S2b). Only two *B*. *napus* accessions, na1 (Ningyou1) and na6 (N02D), produced ROS in response to BcNEP2 (Figures 1e and 2a,b).

**TABLE 2:** Timing of flg22 and BcNEP2 response in accessions of *Brassica* species in the triangle of U using all plants for flg22 but only those that responded to BcNEP2 peptide for calculation of the up‐time (as defined in Figure S1)

Species	Genome	All flg22^a^	Selected flg22^b^	BcNEP2^b^
*B. rapa*	AA	5.54 ± 0.10	5.57 ± 0.12	4.60 ± 0.37
*B. juncea*	AABB	5.67 ± 0.07	5.50	5.00
*B. nigra*	BB	11.08 ± 0.81	10.90 ± 0.88	15.35 ± 1.87
*B. carinata*	BBCC	8.19 ± 0.21	8.15 ± 0.21	11.85 ± 1.06
*B. oleracea*	CC	8.02 ± 0.25	nd	nd
*B. napus*	AACC	6.86 ± 0.17	7.58 ± 0.25	8.17 ± 1.00

Abbreviation: nd, no data.

^a^ Mean (min) ± *SEM* of all lines in this species.

^b^ Mean (min) ± *SEM* of only the lines responding to BcNEP2 in this species.

Individual plants of certain accessions behaved similarly in their response to flg22 but occasionally showed differences in their BcNEP2 response (Figure S2). While plants from fixed foundation sets (e.g., the *B*. *napus* accessions) responded uniformly, the variation in percentage of plants showing any response was larger for commercial seed and landraces, suggesting this is linked to the degree of genetic fixing. The timing of MAMP‐induced ROS production varied within and between species; when tested at 100 nM, there was no difference in timing between flg22‐ and BcNEP2‐induced ROS within most accessions (Table 2, Figure S3).

### 3.2 Induction of  MAMP‐responsive genes in *B*. *napus*

The flg22‐inducible genes identified by RNAseq (Lloyd et al., 2014) were confirmed by qPCR to be induced by flg22 (Figure 2c and Figure S4). There was a 0.5–6‐fold variation in the level of induction between Ningyou1, Ningyou7, N02D and Tapidor compared to Temple in a previous RNAseq study (Figure S5), showing that genes selected from that study can be used to show dynamics of gene induction among varieties. The fungal MAMP chitin also induced ROS in most *B*. *napus* accessions (Figure S6). Most of the PTI marker genes were induced to similar levels by chitin and flg22 at 1 h but to higher levels by chitin at 3 h in each cultivar (Figure S4). We therefore consider *cyp81f2*, *pen2*, *mlo6*, *mlo12* and *cyp706a1* (Table S3) as marker genes for MAMP responses in brassicas.

In N02D, an accession responding to BcNEP2 in the ROS assay, BcNEP2 induced most of the PTI marker genes (Figure 2c). Expression of *cyp81f2*, *pen2*, *mlo6* and *cyp706a1* was significantly induced in both N02D (Figure 2c) and Ningyou1 but not in Ningyou7 and Tapidor, accessions without ROS response to BcNEP2 (Figure S4). Induction of MAMP‐responsive genes was never significant in accessions that did not produce ROS in response to BcNEP2 (Figure S4).

### 3.3 Recognition of NLP sequences in *B. napus* accessions

**FIGURE 3:**
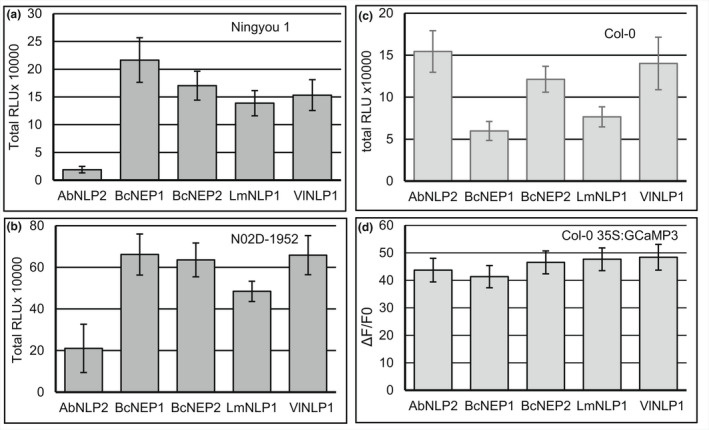
Peptides with MAMP motif from NLPs of the *Brassica* pathogens *Alternaria brassicicola* (AbNLP2), *Botrytis cinerea* (BcNEP1 and BcNEP2), *Leptosphaeria maculans* (LmNLP1) and *Verticillium longisporum* (VlNLP1) are recognized by plants that recognize BcNEP2. Leaf discs of (a) *Brassica napus* na1 (Ningyou1), (b) *B*. *napus* na6 (N02D), (c) *Arabidopsis thaliana* Col‐0 and (d) *A*. *thaliana* Col‐0 expressing 35S:GCaMP3 were challenged with 100 nM of each peptide and reactive oxygen species (ROS) response recorded as relative luminescence units (RLUs) and changes in intracellular [Ca^2+^] recorded as relative changes in fluorescence (Δ*F*/*F*_0_) for 40 min. Bars represent means (±*SEM*) of three individual experiments with eight leaf discs each.

We tested whether *B*. *napus* accessions responding to BcNEP2 would also respond to NLP peptides from other pathogens. We synthesized peptides from *Brassica* pathogens ranging from 24 to 27 amino acids depending on the species (Table 1), covering motifs I and II (Oome et al., 2014). We first tested these for induction of ROS on two *B*. *napus* accessions recognizing BcNEP2 (Ningyou 1 and N02D) and compared them to two nonrecognizing accessions (Tapidor and Ningyou 7). Ningyou1 (Figure 3a) and N02D (Figure 3b) recognized all NLP peptides tested. Responses of similar strength were obtained with NLP peptides from *B*. *cinerea* (BcNEP1 and BcNEP2), *V*. *longisporum* (VlNLP1) and *L*. *maculans* (LmNLP1) whereas AbNLP2 from *A*. *brassicicola* and also HaNLP3 from *H*. *arabidopsidis* induced a weaker response in *B*. *napus* (Figure 3, Figures S7 and S9). As expected, the peptide PccNLP, representing the domain from a type 2 NLP (Table 1), was not recognized in the *B*. *napus* lines tested (Figure S7). Neither Tapidor nor Ningyou7 recognized any of the NLP peptides from the other pathogens (Figure S10). We assessed whether higher concentrations of any NLP could trigger a response in *B*. *oleracea*, which is nonresponsive to BcNEP2, and even 1250 nM was not enough to induce ROS in A12 (Figure S10c). All accessions produced ROS in response to flg22, albeit at different levels, confirming the suitability of our test conditions.

### 3.4 Induction of ROS and elevated [Ca^2+^]_cyt_ in *Arabidopsis* by NLP peptides from *Brassica* pathogens

**FIGURE 4:**
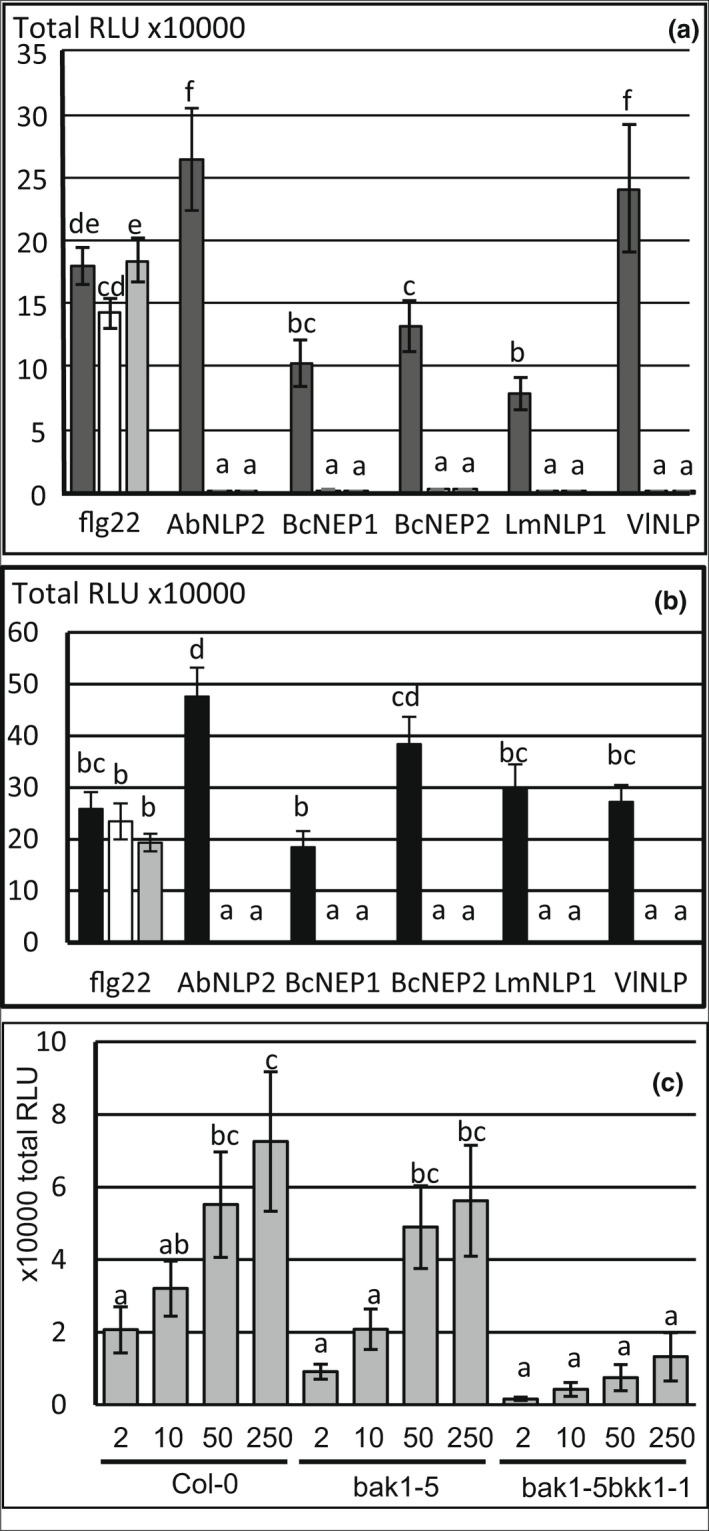
Requirement of (co‐)receptors for recognition of NLP‐MAMP motifs from *Brassica* pathogens in *Arabidopsis*. (a) The wild‐type (WT) Col‐0 (dark grey) and two independent mutant alleles *rlp23‐1* (white) and *rlp23‐2* (light grey) were challenged with 50 nM of each peptide. (b) The WT Col‐0 (dark grey), *sobir1‐12* (white) and *sobir1‐13* (light grey) were challenged with 50 nM of each peptide. (c) The WT Col‐0, single mutant *bak1‐5* and double mutant *bak1‐5 bkk1‐1* were challenged with a concentration range of BcNEP2 (2 to 250 nM). Reactive oxygen species (ROS) production was recorded as relative luminescence units (RLUs) for 40 min. Bars represent means (±*SEM*) of three individual experiments. Bars with different letters are significantly different (*p* < 0.05) according to Fisher's unprotected LSD in an analysis of variance with unbalanced design.

NLP peptides from all *Brassica* pathogens induced ROS in Col‐0 (Figure 3c) that was stronger than that of the previously described HaNLP3 (Figure S7). However, in contrast to *B*. *napus*, AbNLP2 and VlNLP1 induced ROS to a greater extent than BcNEP1, BcNEP2 and LmNLP1 in Col‐0 (Figures 3 and 4) in the majority of assays. As in *B*. *napus*, the magnitude of the ROS response in *Arabidopsis* was greater at higher concentrations of the *Brassica* pathogen NLPs (Figures [Link], [Link] and S12).

All NLP peptides from *Brassica* pathogens induced elevated [Ca^2+^]_cyt_ in Col‐0 expressing 35S:GCaMP3 even stronger than HaNLP3 or flg22 (Figure 3d). Both amplitude and timing of the [Ca^2+^]_cyt_ response were concentration dependent (Figure S8). In *Arabidopsis* Col‐0 lower concentrations of BcNEP2 caused the peaks in both ROS and [Ca^2+^]_cyt_ responses to occur later. At 50 nM the flg22 peaks occurred earlier than the BcNEP2 peaks and returned to background levels faster, with *t*_max_ for ROS at 13.2 ± 0.2 min for flg22 being significantly less than *t*_max_ 19.3 ± 1.0 min for BcNEP2 and the *t*_max_ for [Ca^2+^]_cyt_ at 6.8 ± 0.4 min for flg22 less than the 12.2 ± 0.9 min for BcNEP2 (Figure S8).

In *Arabidopsis Atrlp23* mutants, ROS was induced by flg22 but not by *Brassica* pathogen NLP peptides (Figure 4a and Figure S11). To confirm that recognition of BcNEP2 involves similar co‐receptors as described for HaNLP3 and PpNLP20, we tested *sobir1* and *bak1‐5* and *bak1‐5 bkk1‐1* mutants. Both *sobir1‐12* and *sobir1‐13* mutants did not respond to the *Brassica* NLP peptides at 50 nM (Figure 4b) but did to 50 nM flg22. Both *sobir1* mutants showed a weak but significant ROS response to 1250 nM BcNEP2, although this was still 100× less than in Col‐0 (Figure S12). ROS production by BcNEP2 or other pathogen NLPs was impaired in the double mutant *bak1‐5 bkk1‐1* but the single mutant *bak1‐5* was hardly affected (Figure 4c and Figure S13). Both *bak1‐5* and *bak1‐5 bkk1‐1* were severely affected in flg22 responses (Figure S14).

### 3.5 Identification of *B*. *napus* genes homologous to *Arabidopsis* genes involved in NLP recognition

**FIGURE 5:**
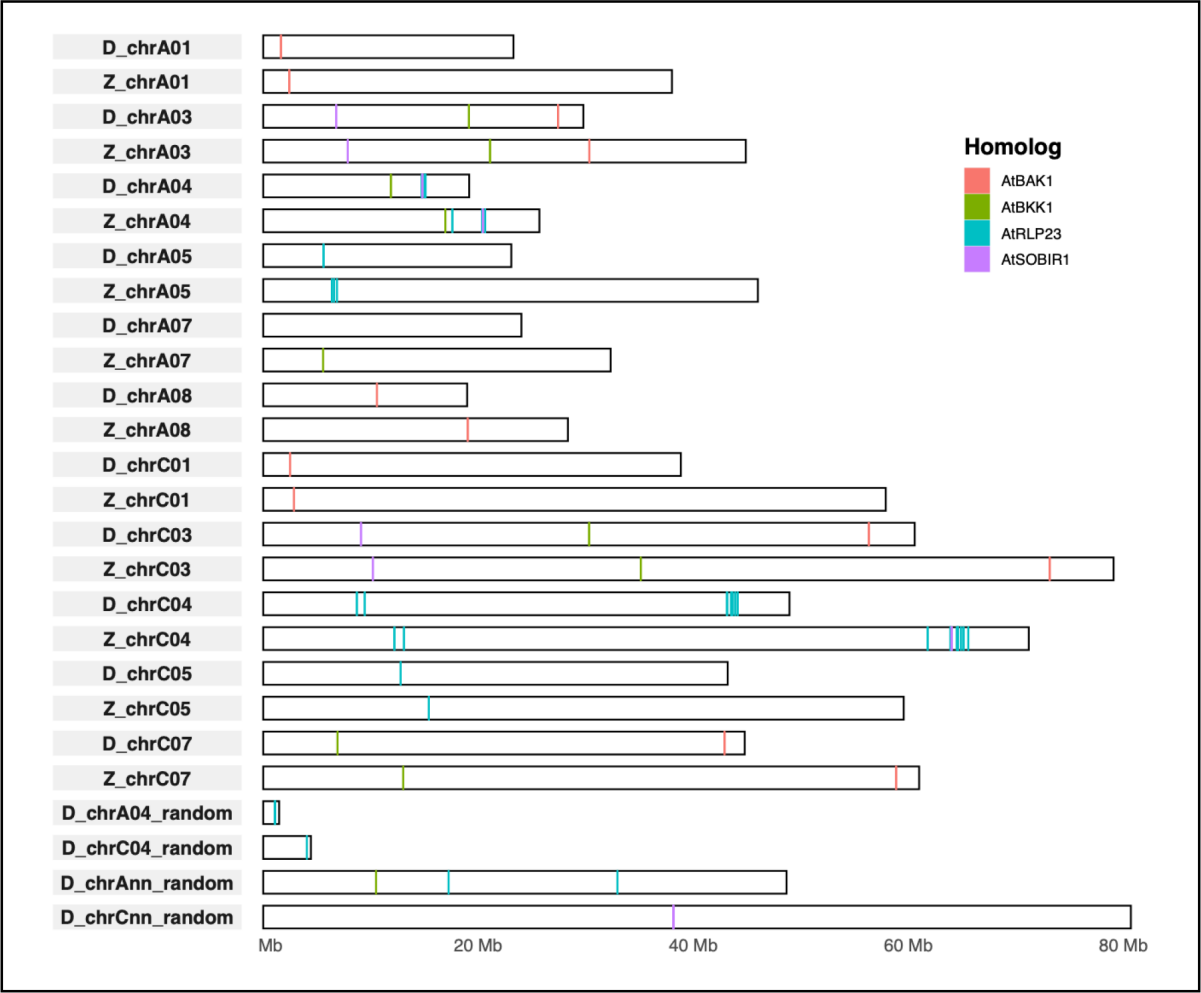
Schematic representation of the presence of gene orthologues involved in NLP recognition. Distribution of *AtSOBIR1* (purple), *AtBAK1* (orange), *AtBKK1* (green) and *AtRLP23* (blue) homologues in *Brassica napus* genomes (Z, Zhongshuang11; D, Darmor‐*bzh*). If genes were detected on contigs not assembled into chromosomes, they are indicated as random with the possible chromosome or Ann and Cnn for contigs mapped to unplaced scaffolds of the A or C genome, respectively. Only chromosomes that have relevant genes on them are displayed as pairs from each reference genome. Length of the chromosomes is shown on *x* axis and markers indicative for size and position are for illustrative purposes. [Colour figure can be viewed at wileyonlinelibrary.com]

The *B*. *napus* accessions Darmor and Zhongshuang11 (ZS11) responded to flg22 by producing ROS but only ZS11 responded to BcNEP2 (Figure S15). We examined the genomes of sequenced *B*. *napus* accessions ZS11 (Song et al., 2020) and Darmor‐*bzh* (Chalhoub et al., 2014) for orthologues of *Arabidopsis* NLP recognition receptor (*AtRLP23*) and co‐receptors *AtSOBIR1*, *AtBAK1* and *AtBKK1* to determine their distribution on the *B*. *napus* A and C genome (Figure 5). The distribution patterns of the receptor and co‐receptor genes along the chromosome were generally similar, although their relative positions were different. All ZS11 genes could be assigned to assembled chromosomes whereas for Darmor‐*bzh* some genes were on contigs that were not assembled into chromosomes (Figure 5).

Darmor, used in this study, should be nearly isogenic with Darmor‐*bzh*, the accession used for the reference genome. We performed whole‐genome comparisons using sequence data from Tudor et al. (2020) with Darmor‐*bzh* to establish whether there were any differences for genes involved with NLP recognition. SNP calling confirmed that there was no difference between Darmor and Darmor‐*bzh* within *AtSOBIR1* and *AtBAK1* orthologues and identified only four nucleotide variants within the regions containing *AtRLP23* and *AtBKK1* orthologues. None of the effects of the variants on the Darmor orthologues was predicted to cause loss‐of‐function or truncation of the protein (Table S4); hence, our further analysis is based on the Darmor*‐bzh* reference genome.

The BLASTN search with *AtSOBIR1* cDNA revealed six orthologues, all with over 80% identity and 90% coverage in both Darmor‐*bzh* and ZS11 (Figure S16; Table S5a), three each on the A and C genome (Figure 5). In both reference genomes the BLASTN search with *AtBAK1* and *AtBKK1* cDNA identified six loci with over 64% homology to *AtBAK1*, and five loci with over 67% homology to *AtBKK1* (Figure S17; Table S5b). One of the BAK1 orthologues from Darmor‐*bzh* reference genome on C03 (*D_Bna*.*BAK1*.*C03*) is located on a poorly assembled contig and therefore appears shorter than its closest homologue in the ZS11 genome (*Z_Bna*.*BAK1*.*C03*).

The BLASTN search of *AtRLP23* cDNA sequence on both *B*. *napus* reference genomes revealed 22 and 24 homologues for Darmor‐*bzh* and ZS11, respectively (Figure 5 and Figure S18; Table S5c). Overall numbers between A and C genome were similar, with nine A and 13 C genome orthologues for Darmor‐*bzh* and nine A and 15 C genome orthologues for ZS11. Orthologues located within the same clusters for both genomes were within the same clades of the phylogenetic tree (Figures [Link], [Link], and S18).

### 3.6 Effect of mutations affecting recognition of NLPs on resistance to *B*. *cinerea* in *Arabidopsis* and *B*. *napus*

Both *Bcnep1* and *Bcnep2* were expressed by *B*. *cinerea* during infection of *B*. *napus* and *A*. *thaliana* (Figure S19), with *Bcnep2* higher than that of the known elicitor *Bcxyn11A* (Noda et al., 2007). Thus, they might be available for recognition and induction of PTI upon *B*. *cinerea* infection.

**FIGURE 6:**
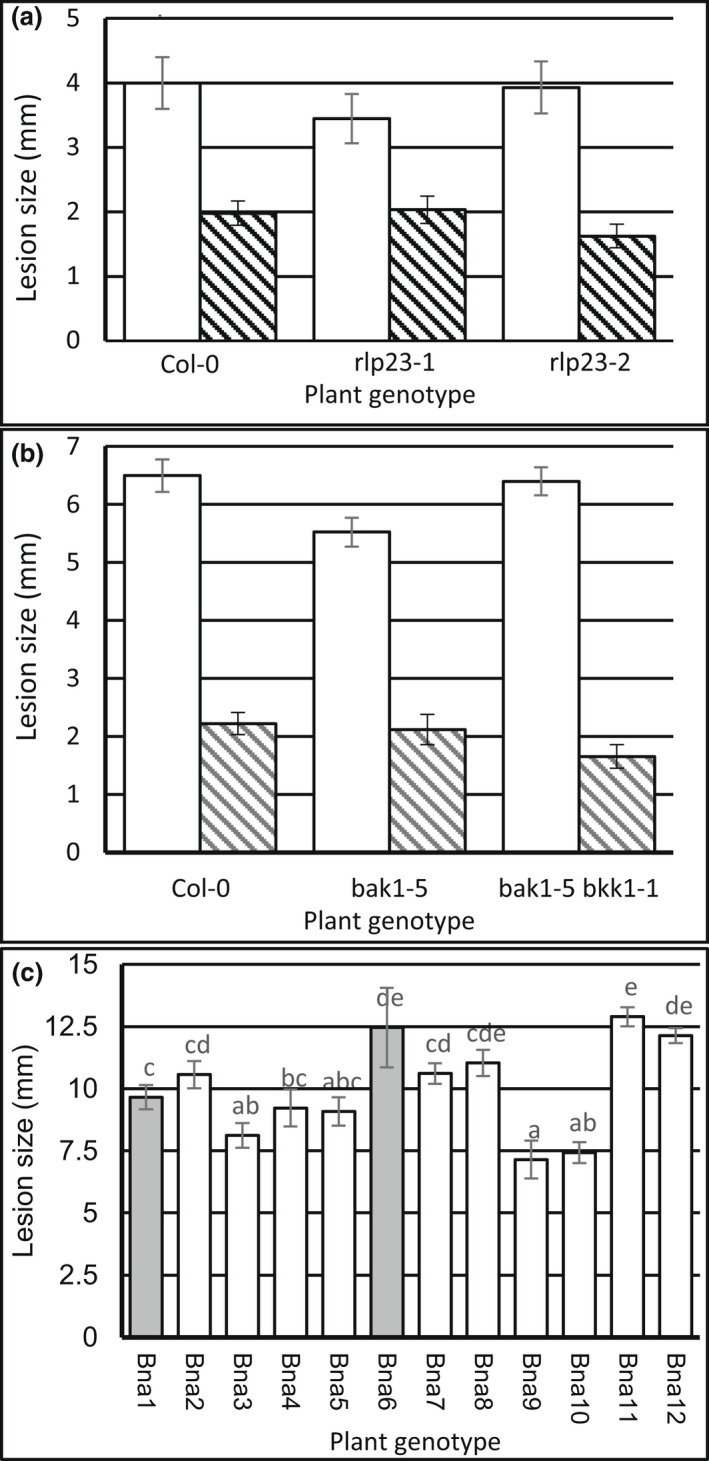
NLP recognition has no major effect on *Botrytis cinerea* susceptibility. (a) Five leaves per plant of *Arabidopsis* wild‐type (WT) Col‐0 and rlp23 mutants *rlp23‐1 and rlp23‐2* were pairwise inoculated with droplets of *B*. *cinerea* B05.10 (white) and ΔBctrB4 (striped); lesion size (diameter) was measured at 3 days postinoculation (dpi). (b) Five leaves per plant of *Arabidopsis* WT Col‐0 and SERK mutants *bak1‐5* and *bak1‐5 bkk1‐1* were pairwise inoculated with droplets of *B*. *cinerea* B05.10 (white) and ΔBctrB4 (striped); lesion size (diameter) was measured at 3 dpi. Bars represent mean (±*SEM*) lesion size (diameter) of three experiments with at least eight plants each. (c) Leaf discs of *Brassica napus* accessions that do (na1 [Ningyou1] and na6 [N02D]; dark grey) or do not (white) recognize BcNEP2 were inoculated with agar plugs of *B*. *cinerea* B05.10 and measured 2 dpi. Bars represent mean (±*SEM*) lesion size (diameter) of three experiments with 16 leaf discs each. Bars marked with different letters are significantly different (*p* < 0.05) according to Fisher's unprotected LSD in an analysis of variance with unbalanced design.

To test if the receptor or co‐receptors involved in NLP recognition contribute to the moderate resistance observed in *Arabidopsis* Col‐0 (Stefanato et al., 2009; Zhang et al., 2016), we compared infection by *B*. *cinerea* on wild‐type Col‐0 with *rlp23*, *bak1‐5* and *bak1‐5 bkk1‐1* mutants. Neither the wild‐type B05.10 nor the camalexin‐sensitive mutant ΔBcatrB4 was more infectious on *rlp23‐1* and *rlp23‐2* (Figure 6a) or on *bak1‐5* or *bak1‐5 bkk1‐1* (Figure 6b). The 12 *B*. *napus* accessions, including NLP‐recognizing Ningyou1 and N02D, were inoculated with agar plugs of *B*. *cinerea*. The two accessions that recognize NLPs were neither more nor less susceptible to *B*. *cinerea* than other accessions (Figure 6c).

## 4 DISCUSSION

Our study presents the first comprehensive investigation of NLP recognition within all six *Brassica* species from the triangle of U. Interestingly, none of the 79 *B*. *oleracea* (CC genome) accessions tested responded to BcNEP1 whereas several *B*. *rapa* (AA genome) and *B*. *nigra* (BB genome) accessions did respond. Surprisingly, the results contrast with *Arabidopsis* in which only 3 of 135 accessions did not respond to NLPs (Albert et al., 2015). The ROS response to flg22 was not much faster than to BcNEP2 (Table 2) in most *Brassica* varieties, except some *B*. *carinata* and *B*. *nigra* accessions that were particularly slow for BcNEP2 response. This contrasts *Brassica* with *Arabidopsis* where RLK‐mediated responses are considered faster than RLP responses (Wan et al., 2019), demonstrating the importance of using multiple varieties when translating research from model plants to crops.

The highly conserved amino acids of MAMP motif I (Oome et al., 2014) are nearly all identical in the tested NLPs but there is variation in the adjacent amino acid AbNLP2, which induced the weakest response of all fungal NLPs in *B*. *napus* but not *Arabidopsis* and has a threonine instead of arginine on position 20 of the consensus sequence. Thus, in contrast to *Arabidopsis*, other amino acids besides the highly conserved ones could affect the strength of responses in *Brassica* species. This rare variant only occurred in another 3 of 500 NLP sequences, two of which are from species pathogenic on *B*. *rapa*, which might limit recognition by RLPs present in *Brassica* host plants.

The gene expression analysis after MAMP treatment in *B*. *napus* (Lloyd et al., 2014) identified genes induced by multiple MAMPs, including BcNEP2 peptide, of which some were separately identified in *Arabidopsis* (Bjornson et al., 2021). One of these is CYP81F2, which corresponds to the horizontal resistance locus BLMR2 and is induced by *L*. *maculans*, potentially linking our expression data with QDR in *B*. *napus* (Zhang et al., 2021).

*Bcnep1* and *Bcnep2* from *B*. *cinerea* were expressed in *Arabidopsis* and *B*. *napus* at 24 hpi, corroborating previous investigations on tomato, lettuce and *Arabidopsis* (Arenas et al., 2010; Emmanuel et al., 2018; Ono et al., 2020). According to Emmanuel et al. (2018), there is no correlation between the expression levels of *Bcnep1* and the appearance of infections with or without symptoms when comparing *Arabidopsis* and lettuce, which both recognize NLPs (Bohm et al., 2014). However, the two *B*. *napus* cultivars that responded to BcNEP2 were not more resistant to *B*. *cinerea* than nonresponding cultivars, neither were the *rlp23* mutants in *Arabidopsis* more susceptible.

The *Brassica* and *Arabidopsis* data together suggest that recognition of NLPs is not sufficient for resistance to *B*. *cinerea*. This is consistent with the observation that *Arabidopsis* basal susceptibility to downy mildew is not affected in *rlp23* mutants (Albert et al., 2015). The *Arabidopsis* results contrast with those of Ono et al. (2020) in which *rlp23* mutants were more susceptible to *B*. *cinerea* than wild‐type plants. However, in this case lesion diameter was measured after 7 days whereas our measurements were taken after 72 h, consistent with previously published procedures (Stefanato et al., 2009; Zhang et al., 2014). Ono et al. (2020) also report that growth of *B*. *cinerea* was not significantly different between the wild type and *rlp23* mutants at 6 and 16 h but was at 12 h. Together, these data indicate the difference in lesion size between *rlp23* mutants and wild‐type *Arabidopsis* could be influenced by virulence of the fungal isolate or other factors affecting this such as spore density and environmental conditions. The lack of correlation between NLP recognition and resistance to *B*. *cinerea* in Brassicaceae could also be explained by the growth conditions and the timing of assessments. Recognition of pathogens relies on more than one MAMP so the loss of one MAMP–PRR combination may not critically reduce the plant defence response. It has been demonstrated that RLP42 can recognize polygalacturonases from *B*. *cinerea* (Zhang et al., 2014) and mounts a stronger immunity response than RLP23 (Wan et al., 2019), explaining why loss of NLP recognition does not cause dramatic losses in disease symptoms in Col‐0. Also, because resistance to pathogens like *B*. *cinerea* is multigenic (Zhang et al., 2016) and the 12 *B*. *napus* cultivars have different genetic backgrounds, variation in factors other than NLP recognition might be so large that it masks the effects of recognition of this elicitor.

The type 1 NLPs from various fungal pathogens are likely to be recognized by the same mechanism in all of the *Brassica* species because the cultivars tested responded either to all or none of the NLPs, and response was lost in the *Arabidopsis rlp23*, *sobir1* and *bak1‐5 bkk1‐1* mutants. This suggests that all peptides with the same conserved motifs are recognized by the same receptor in *Arabidopsis*, RLP23 (Albert et al., 2015), and requires the co‐receptors SOBIR1, BAK1 and BKK1 to varying degrees. Perception of *Brassica* pathogen NLPs in *Arabidopsis* was more impaired in the *bak1‐5 bkk1‐1* double mutant than in the single *bak1‐5* mutant, suggesting that both SERKs contribute to NLP‐induced signalling. Under our conditions the loss of response in *bak1‐5* on NLP‐peptide recognition was small and not significant at any concentration between 2 and 250 nM while the effect on flg22 response was highly significant. This is partially in concordance with Wan et al. (2019), who reported that the ROS response to 500 nM nlp20 in *bak1‐5* was significantly different from the wild type but the impact was over two orders of magnitude smaller than on flg22 response. AtRLP42 activates defence responses via similar pathways (Zhang et al., 2014), including BAK1 and SOBIR1 (Albert et al., 2015). Signalling through these co‐receptors has at the same time effects supporting and restricting infection by a necrotroph, for example through induction of cell death (Zhang et al., 2014), and induction of indole defence compounds (Zhang et al., 2013). The balance between these effects might depend on environmental factors such as lighting and explains some of the differences observed between this study and some, but not all, other laboratories (Wan et al., 2019; Zhang et al., 2013, 2014).

We expect other Brassicaceae to recognize NLPs through an *AtRLP23* orthologue, for which there are multiple candidates in *B*. *napus*. Annotated *RLP* genes with a homology over 60% to *AtRLP23* are unequally distributed over the *B*. *napus* chromosomes and most are found in clusters on C04 and A04. This follows the pattern of clustered distribution described for all *RLP*s and the whole LRR family in Brassicaceae (Stotz et al., 2018) that is probably caused by rapid dynamics through duplication (Yang et al., 2021). The distribution pattern as paired homeologues over A and C genomes of these potential *AtRLP23* orthologues, but also of *BKK1*, occurs less frequently than for *BAK1* and *SOBIR1* because for the latter we find matching homeologue pairs on the A and C genome for both Darmor‐*bzh* and ZS11. This result for *AtRLP23* orthologues is in concordance with observations by Stotz et al. (2018) that of 184 RLPs annotated in Darmor‐*bzh* only 58 were considered to be direct homeologue pairs. This suggests that *RLP23* genes have diverged between the A and C genome. Because no BcNEP2 responsiveness was detected in any *B*. *oleracea* accession, this would imply functional loss of the NLP receptor in the C‐genome progenitor. This is supported by the fact that NLP responsiveness is only observed in species that have at least one A or B genome. However, it is very difficult to predict which one would be the functional *RLP23* copy because the genes with the highest homology have direct homologues on the *B*. *napus* A and C genome as well as in the sequenced *B*. *rapa* and *B*. *oleracea* genomes (https://plants.ensembl.org/). Considering the complexity of the allopolyploid genomes, further genetic and experimental analyses are required to determine any likely candidate for a functional *RLP23* homologue in *B*. *napus*.

Whole genome duplication in polyploids is a major factor in plant diversification because the introduced genetic redundancy enables the evolution of novel gene functions and interactions. Polyploid *Brassica* genomes are subject to frequent rearrangement and gene loss, and there can be marked differences between accessions even of the same species. The expansion and rearrangement of genomes in the brassicas develops interesting evolutionary questions about which genes are retained and lost. Our investigation demonstrates that while the mechanisms involved with NLP recognition and responses might be similar to *Arabidopsis*, the genomic inventory of immune receptors is much more complex in polyploid *Brassica* species. Also, our results highlight challenges in comparing phenotypic traits in *Arabidopsis* with those in crops, even within the Brassicaceae, with complex genetic backgrounds and variable growing conditions. Further investigation into the environmental effects of phenotypes and impact of receptor complexity on the immune system is required to translate research more efficiently from model plants to improve disease resistance traits in polyploid crop species.

## CONFLICT OF INTEREST

We declare no conflict of interest or connection to financial stakeholders that benefit from this research.

## Supporting information


Figure S1

Figure S2

Figure S3

Figure S4

Figure S5

Figure S6

Figure S7

Figure S8

Figure S9

Figure S10

Figure S11

Figure S12

Figure S13

Figure S14

Figure S15

Figure S16

Figure S17

Figure S18

Figure S19

Table S1

Table S2

Table S3

Table S4

Table S5

## Data Availability

The data that support the findings of this study are available in public repositories, as stated in the Materials and Methods, and in the supplementary material of this article.
