# Supplementary material for: Necrosis and ethylene‐inducing‐like peptide patterns from crop pathogens induce differential responses within seven brassicaceous species
Source: Plant Pathol. 2022 Aug 5;71(9):2004–16. doi: 10.1111/ppa.13615 (PMC9804309; doi:10.1111/ppa.13615)
Supplement: Supplementary file 1 — Figure S1 [file PPA-71-2004-s006.pdf]

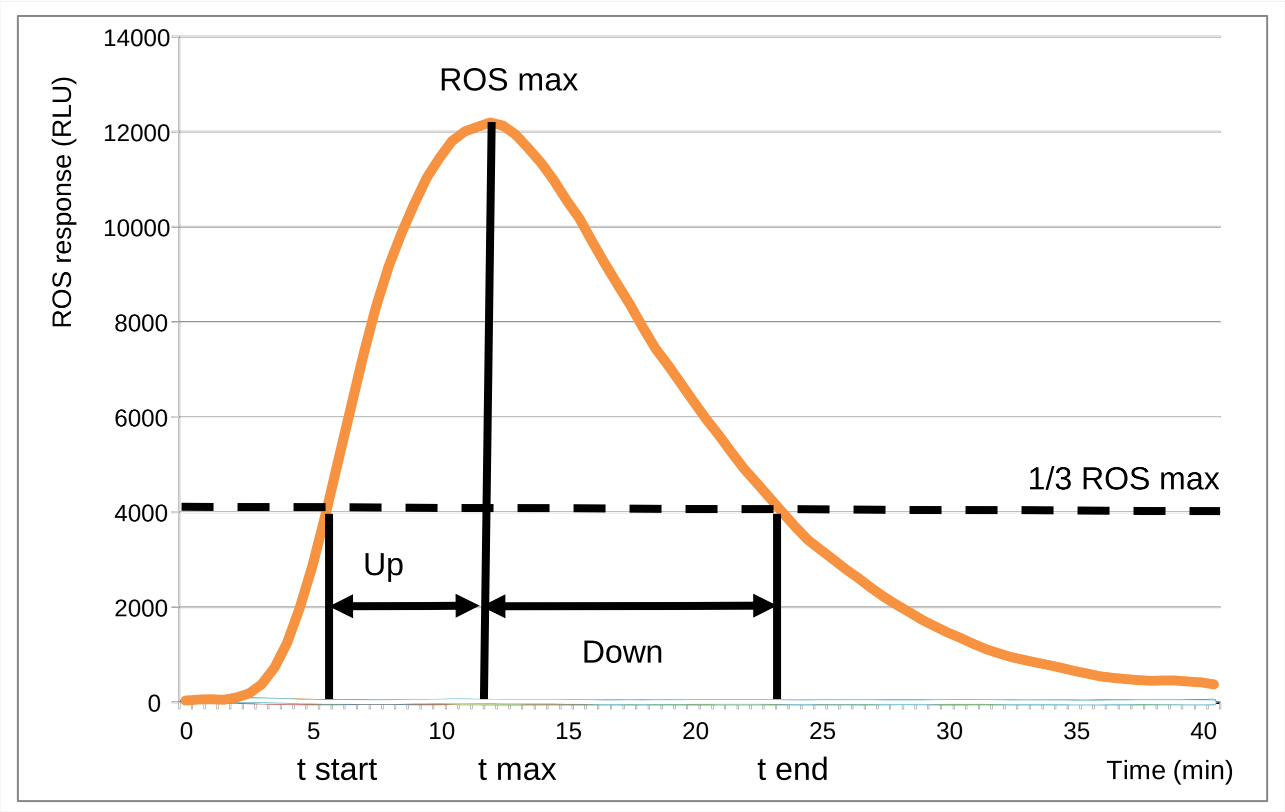

Figure S1

Breakdown of timings at a typical transient ROS-curve. The absolute timing of events relative to the moment of PAMPs being added varies slightly from plate to plate due to differences in handling speed and the machine performing internal controls. Therefore, we chose the relative timing within each run as a parameter to compare timings between lines. We determined the level (ROS max) and timing (t max) at the top of the response peak and calculated the moment the curve first went up to 1/3 of the maximum response as the moment the response unequivocally had started (t start) and went back down to 1/3 of the maximum as the end of the peak (t end). "Up time" as a measure of the speed of response was calculated as the t max minus t start.
