# Supplementary material for: Necrosis and ethylene‐inducing‐like peptide patterns from crop pathogens induce differential responses within seven brassicaceous species
Source: Plant Pathol. 2022 Aug 5;71(9):2004–16. doi: 10.1111/ppa.13615 (PMC9804309; doi:10.1111/ppa.13615)
Supplement: Supplementary file 2 — Figure S2 [file PPA-71-2004-s017.pdf]

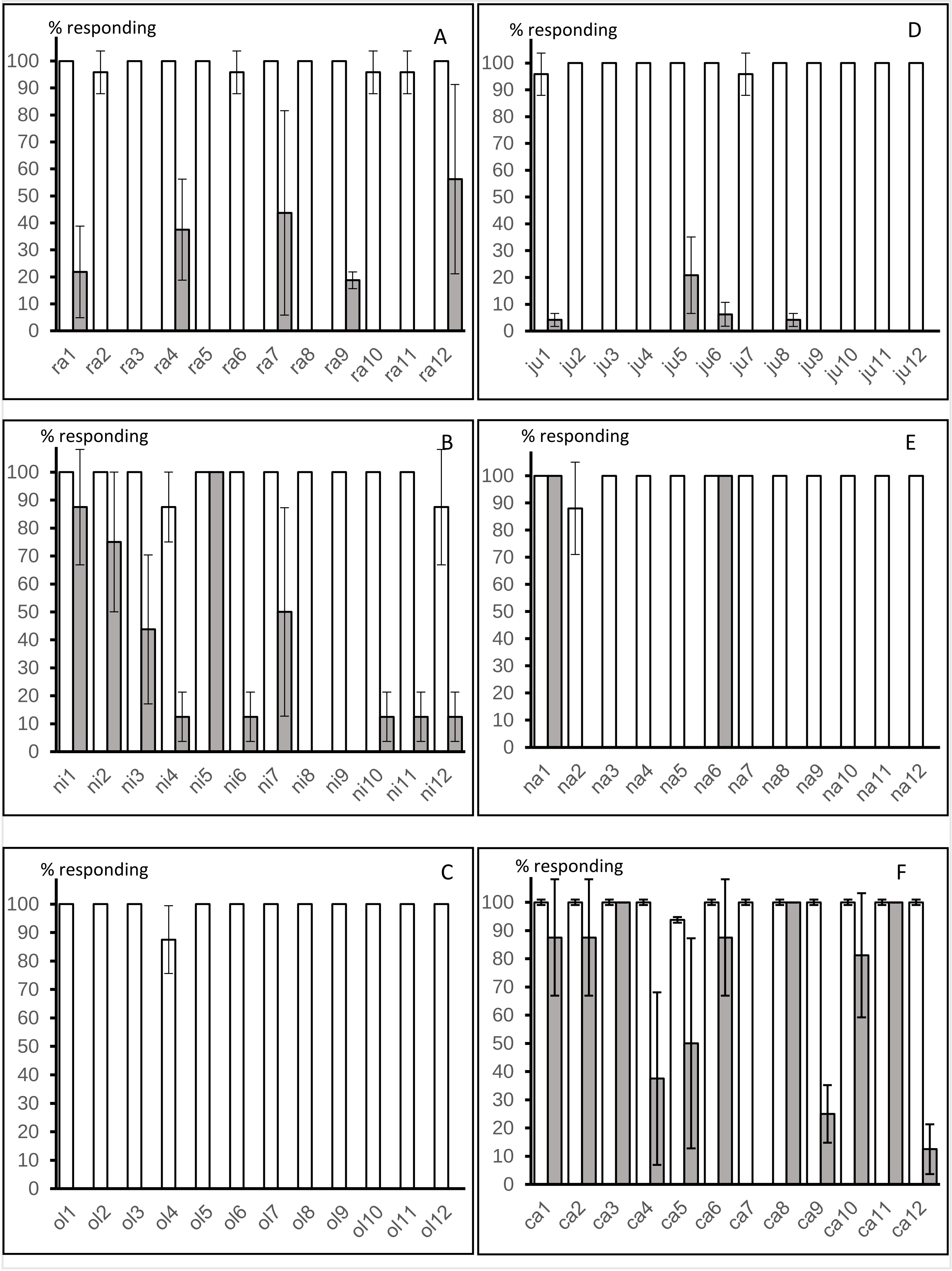

Figure S2

Percentage of leafdiscs responding to flg22 (white) and BcNEP2 peptide (grey) in each accession of Brassica species in the triangle of U. (A) *B. rapa* (AA), (B) *B. nigra* (BB), (C) *B. oleracea* (CC), (D) *B. juncea* (AABB), (E) *B. napus* (AACC), (F) *B. carinata* (BBCC). Bars represent means (+/- SEM) of 3 experiments with 8 leafdiscs each.
