# Supplementary material for: Necrosis and ethylene‐inducing‐like peptide patterns from crop pathogens induce differential responses within seven brassicaceous species
Source: Plant Pathol. 2022 Aug 5;71(9):2004–16. doi: 10.1111/ppa.13615 (PMC9804309; doi:10.1111/ppa.13615)
Supplement: Supplementary file 3 — Figure S3 [file PPA-71-2004-s010.pdf]

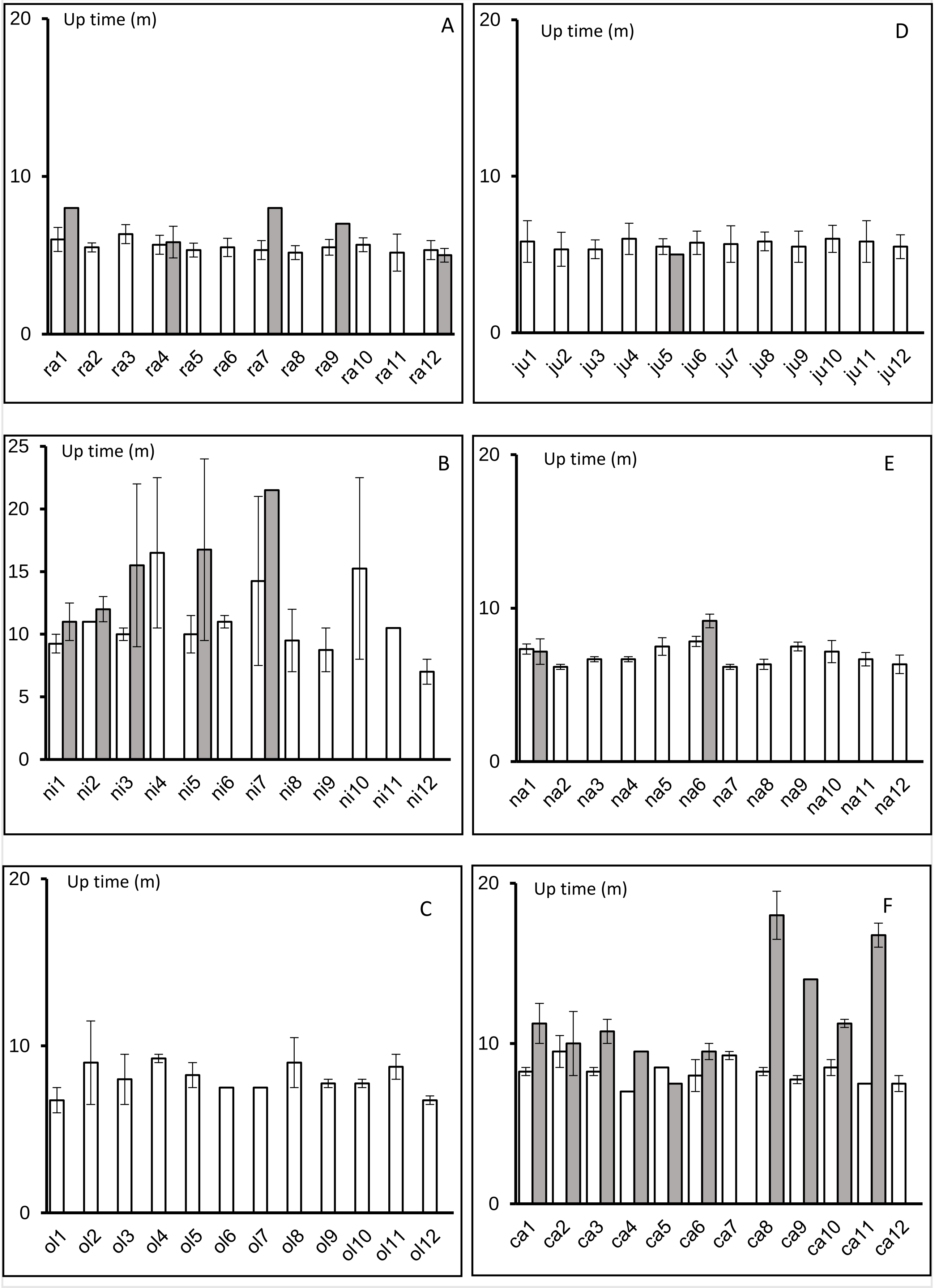

Figure S3

Timing of flg22 and BcNEP2 response in each accession of Brassica species in the triangle of U in response to flg22 (white) and BcNEP2 (grey) for calculation of the “up time” (as defined in Figure S1). *(A) B. rapa* (AA), (B) *B. nigra* (BB), (C) *B. oleracea* (CC), (D) *B. juncea* (AABB), (E) *B. napus* (AACC), (B) (F) *B. carinata* (BBCC). Bars represent means of 3 experiments (+/- SEM).
