# Supplementary material for: Necrosis and ethylene‐inducing‐like peptide patterns from crop pathogens induce differential responses within seven brassicaceous species
Source: Plant Pathol. 2022 Aug 5;71(9):2004–16. doi: 10.1111/ppa.13615 (PMC9804309; doi:10.1111/ppa.13615)
Supplement: Supplementary file 4 — Figure S4 [file PPA-71-2004-s016.pdf]

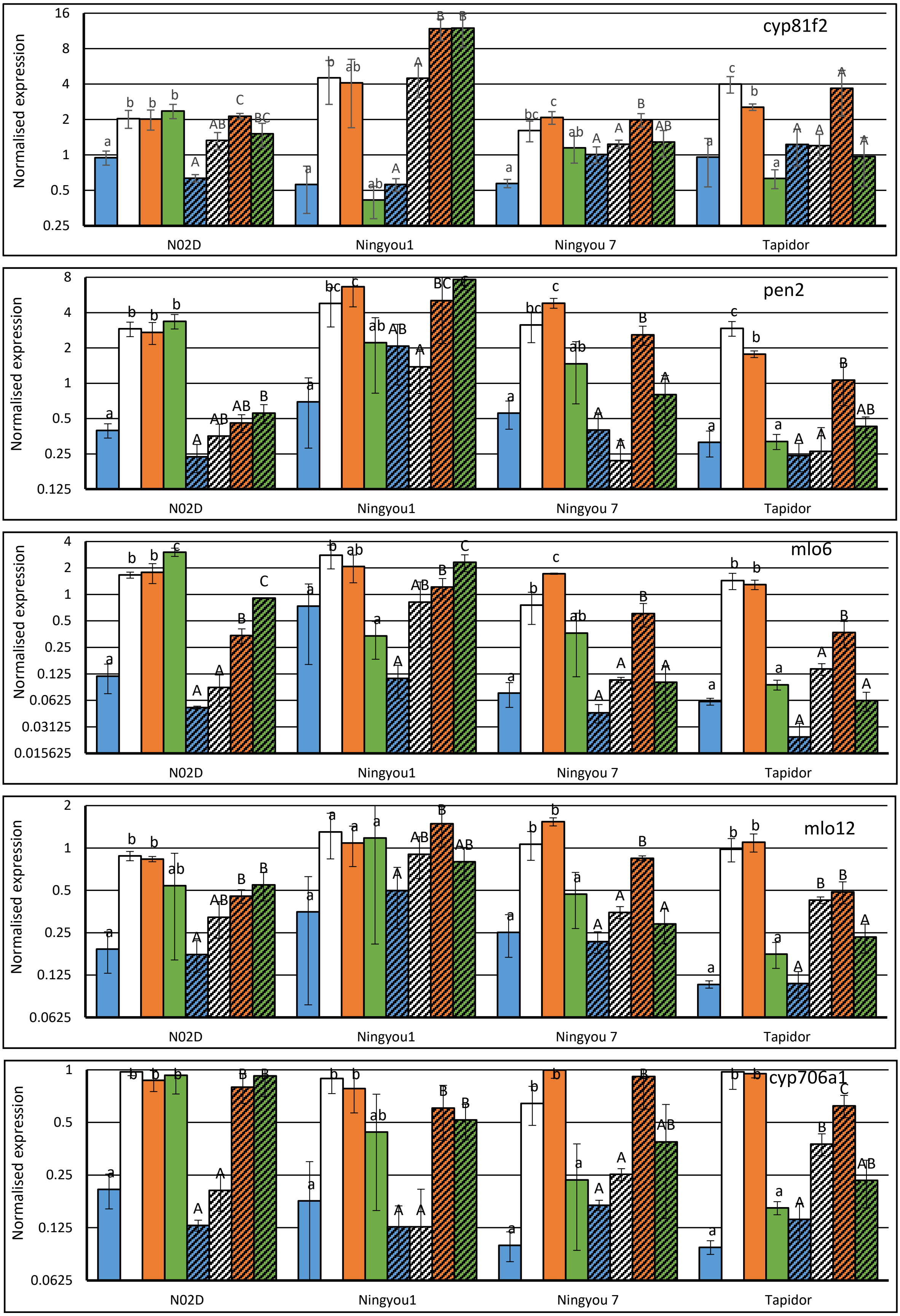

Figure S4

Gene-expression in four *B. napus* cultivars N02D (na6), Ningyou1 (na1), Ningyou7 (na8) and Tapidor (na3) normalised to *BnEF1a*. Pre-infiltrated leaf discs were treated with water (blue), 100nM flg22 (white), 500mg/l chitin (orange) or 100nM BcNEP2 peptide (green) for 1h (no pattern, small letters) or 3h (striped, capitals). Bars represent the mean (+/- SEM) of at least 3 individual treatments and RNA extractions. Within each cultivar the four treatments per time-point were compared by ANOVA and significant differences (p<0.05) between treatments at the same timepoint according to Fishers LSD are indicated with different letters.
