# Supplementary material for: Necrosis and ethylene‐inducing‐like peptide patterns from crop pathogens induce differential responses within seven brassicaceous species
Source: Plant Pathol. 2022 Aug 5;71(9):2004–16. doi: 10.1111/ppa.13615 (PMC9804309; doi:10.1111/ppa.13615)
Supplement: Supplementary file 5 — Figure S5 [file PPA-71-2004-s004.pdf]

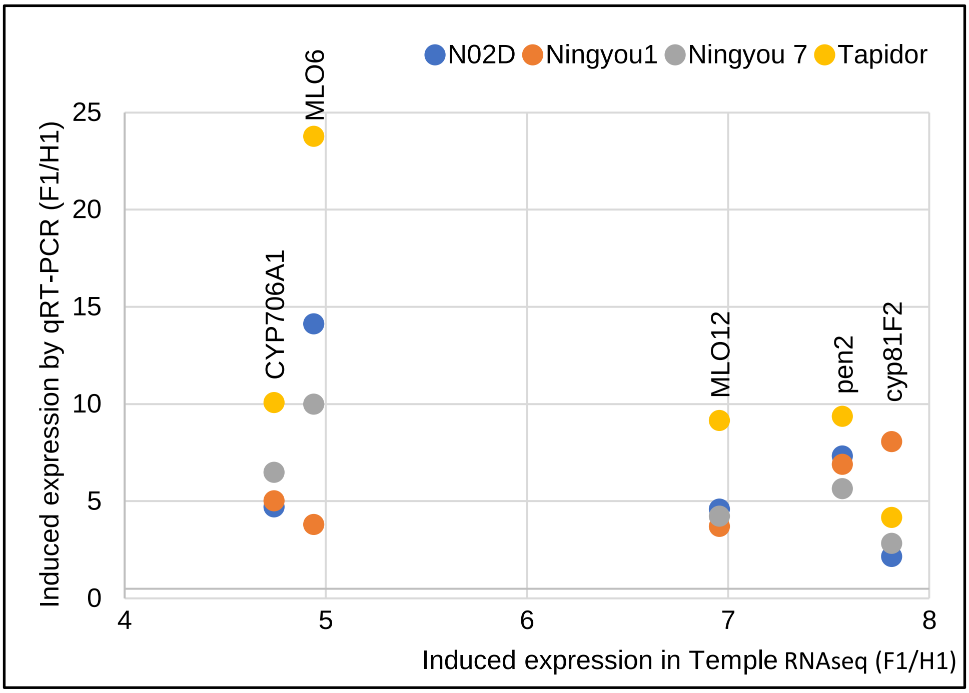

Figure S5
Comparison between induction levels of PAMP-induced genes (PIGs) in the Temple RNAseq experiment (horizontal axis, Lloyd *et al*. 2014) and the 4 cultivars used for qRT-PCR in this study. Induction (F1/H1) was calculated as the normalised expression (relative to *BnEF1a*) after flg22 treatment for 1 h (F1) divided by normalised expression after mock treatment with H_2O for 1h (H1).
