# Supplementary material for: Necrosis and ethylene‐inducing‐like peptide patterns from crop pathogens induce differential responses within seven brassicaceous species
Source: Plant Pathol. 2022 Aug 5;71(9):2004–16. doi: 10.1111/ppa.13615 (PMC9804309; doi:10.1111/ppa.13615)
Supplement: Supplementary file 6 — Figure S6 [file PPA-71-2004-s001.pdf]

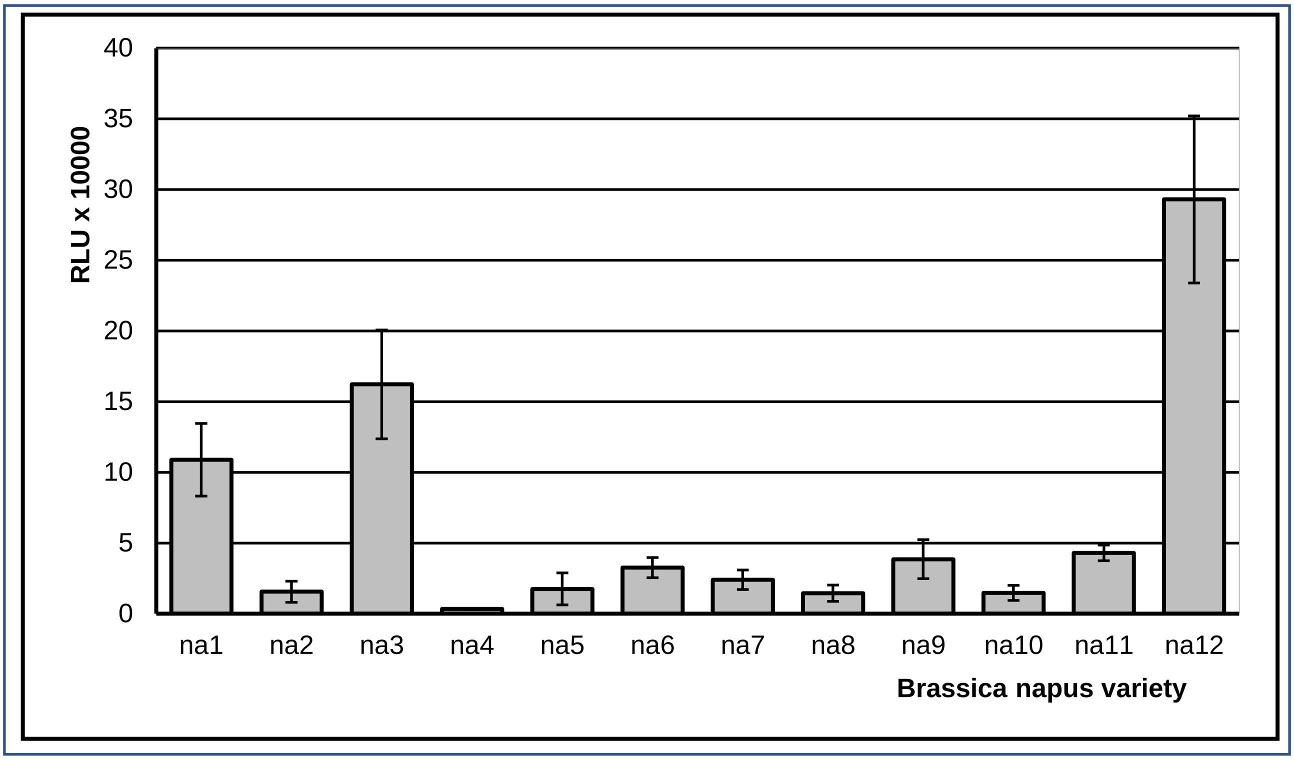

Figure S6
Recognition of fungal MAMPs in *Brassica napus*. Total ROS response of twelve cultivars of *B. napus* treated for 40 min with 500 mg/l chitin measured as relative light units (RLU). Bars display the mean (+/- SEM) of 3 independent experiments with 4 plants each.
