# Supplementary material for: Necrosis and ethylene‐inducing‐like peptide patterns from crop pathogens induce differential responses within seven brassicaceous species
Source: Plant Pathol. 2022 Aug 5;71(9):2004–16. doi: 10.1111/ppa.13615 (PMC9804309; doi:10.1111/ppa.13615)
Supplement: Supplementary file 7 — Figure S7 [file PPA-71-2004-s007.pdf]

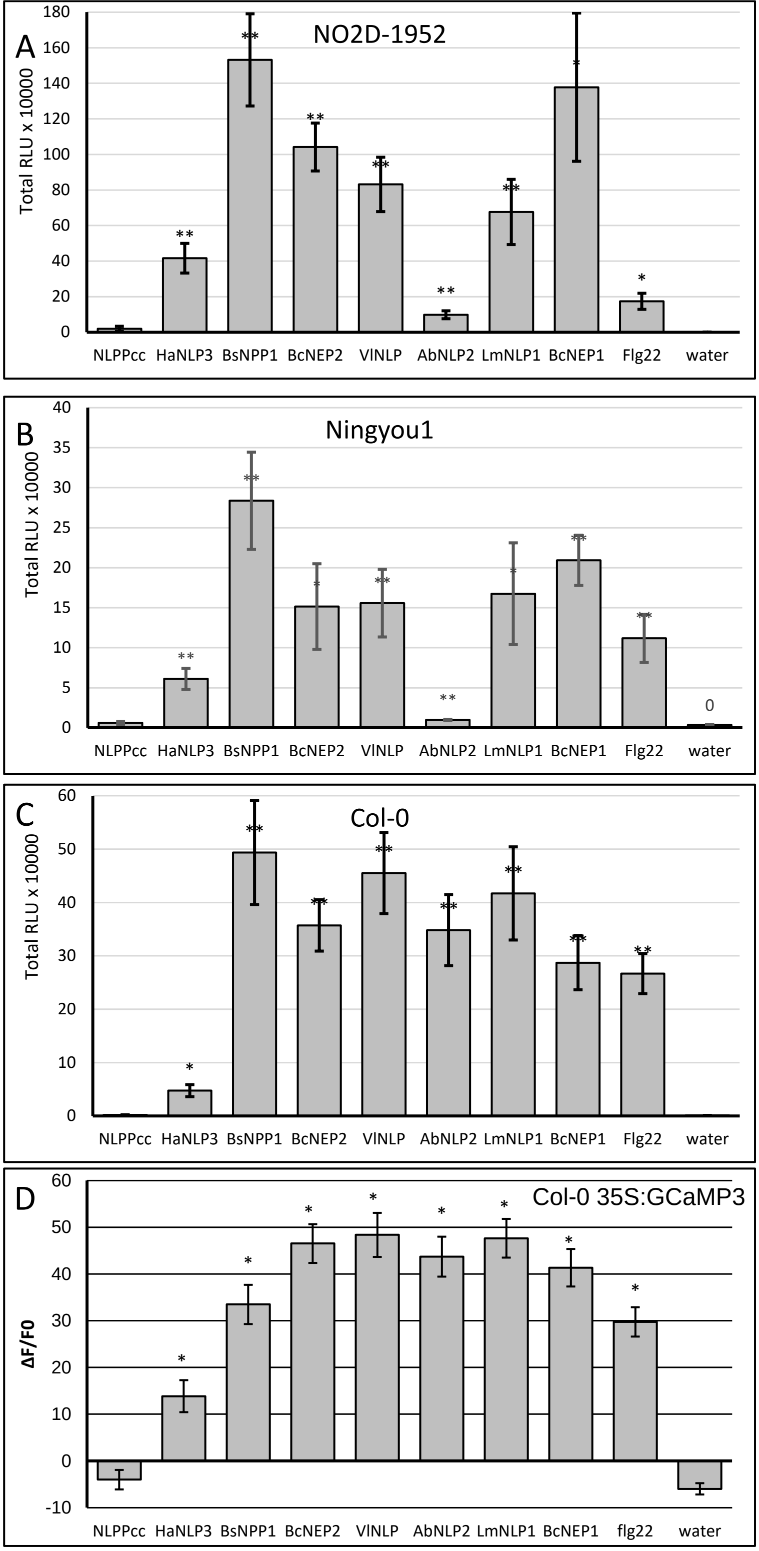

Figure S7. Only MAMP motifs from type 1 NLPs, not type 2, are recognised by responsive *Brassicaceae.* In contrast to the type 2 NLP from *Pectobacterium carotovorum* (NLPPcc), the MAMP motif from the previously described NLPs of *Hyaloperonospora arabidopsidis* (HaNLP3) and *Bacillus subtilis* (BsNLP) as well as the Brassica pathogens *Alternaria brassicicola* (AbNLP2), *Botrytis cinerea* (BcNEP1 and BcNEP2), *Leptosphaeria maculans* (LmNLP1) and *Verticillium longisporum* (VlNLP1) are recognised by plants that recognise BcNEP2, albeit with variable strength. Leaf discs of (A) *Brassica napus* na1 (Ningyou1), (B) *B. napus* na6 (N02D), (C) *Arabidopsis thaliana* Col-0 and (D) *A. thaliana* Col-0 expressing 35S:GCaMP3 were challenged with 50 nM of each peptide and ROS-response recorded as relative luminesce units (RLU) and changes in intracellular Ca^{2+} recorded as relative changes in fluorescence ($\Delta F/F_0$) for 40 min. Bars represent mean (+/-SEM) of 3 experiments with 8 leafdiscs per treatment each. Significant differences from the water treatment were determined by Students' t-test and indicated by * (P<0.05) or ** (P<0.01).
