# Supplementary material for: Necrosis and ethylene‐inducing‐like peptide patterns from crop pathogens induce differential responses within seven brassicaceous species
Source: Plant Pathol. 2022 Aug 5;71(9):2004–16. doi: 10.1111/ppa.13615 (PMC9804309; doi:10.1111/ppa.13615)
Supplement: Supplementary file 8 — Figure S8 [file PPA-71-2004-s002.pdf]

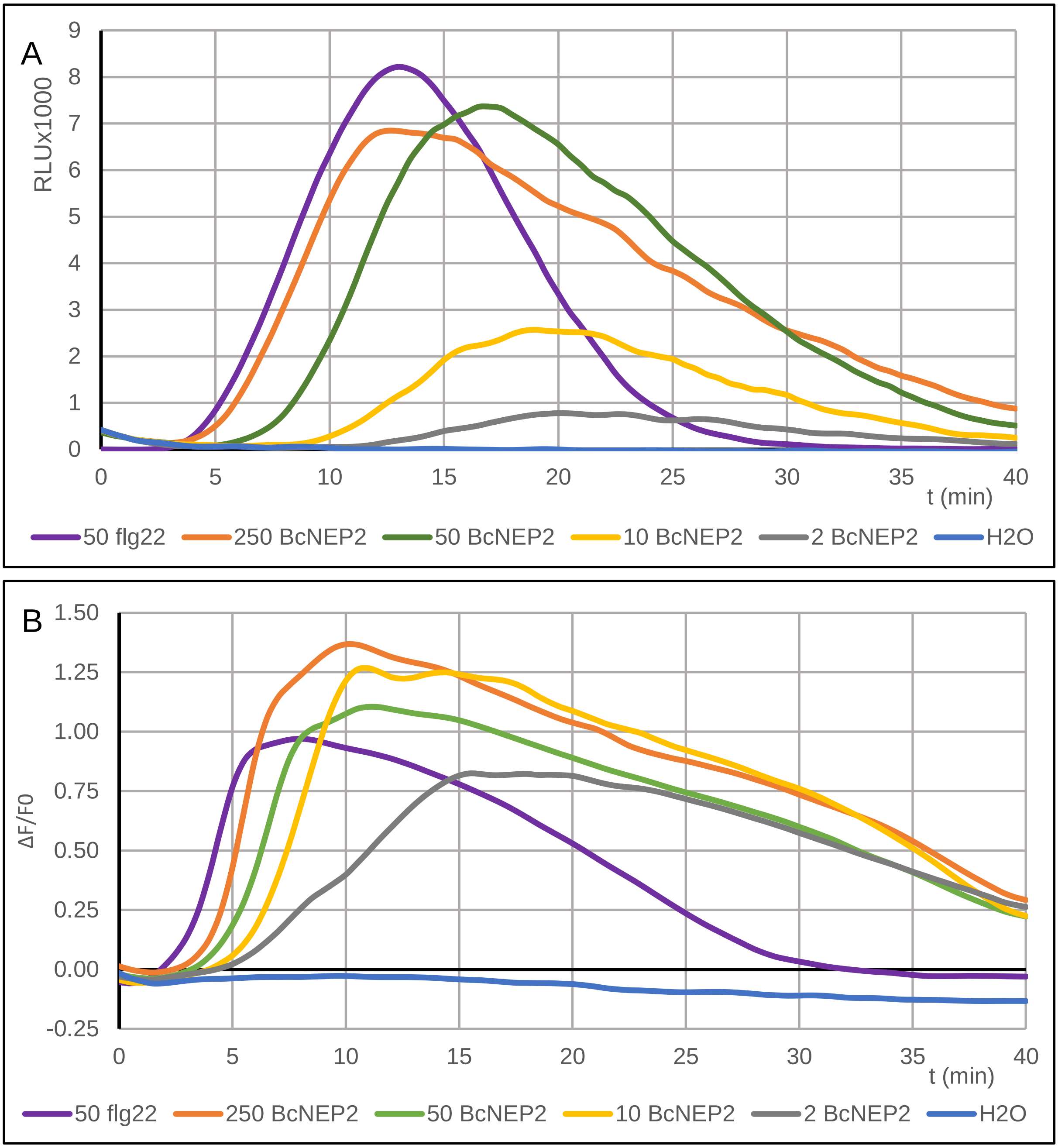

Figure S8. Transient responses to MAMP peptides in Arabidopsis for (A) extracellular ROS production and (B) intracellular Ca^{2+}concentrations. Leafdiscs of Col-0 (A) and Col-0 expressing 35S:GCaMP3 (B) were treated with water, 50 nM flg22 or 2, 10, 50 and 250 nM of BcNEP2 for 40 min and luminescence (RLU; A) and fluorescence (expressed as $\Delta F/F_0$; B) measured every 30s. Curves represent the mean of 8 leafdiscs in one representative experiment which was repeated 3 times with similar results.
