# Supplementary material for: Necrosis and ethylene‐inducing‐like peptide patterns from crop pathogens induce differential responses within seven brassicaceous species
Source: Plant Pathol. 2022 Aug 5;71(9):2004–16. doi: 10.1111/ppa.13615 (PMC9804309; doi:10.1111/ppa.13615)
Supplement: Supplementary file 9 — Figure S9 [file PPA-71-2004-s023.pdf]

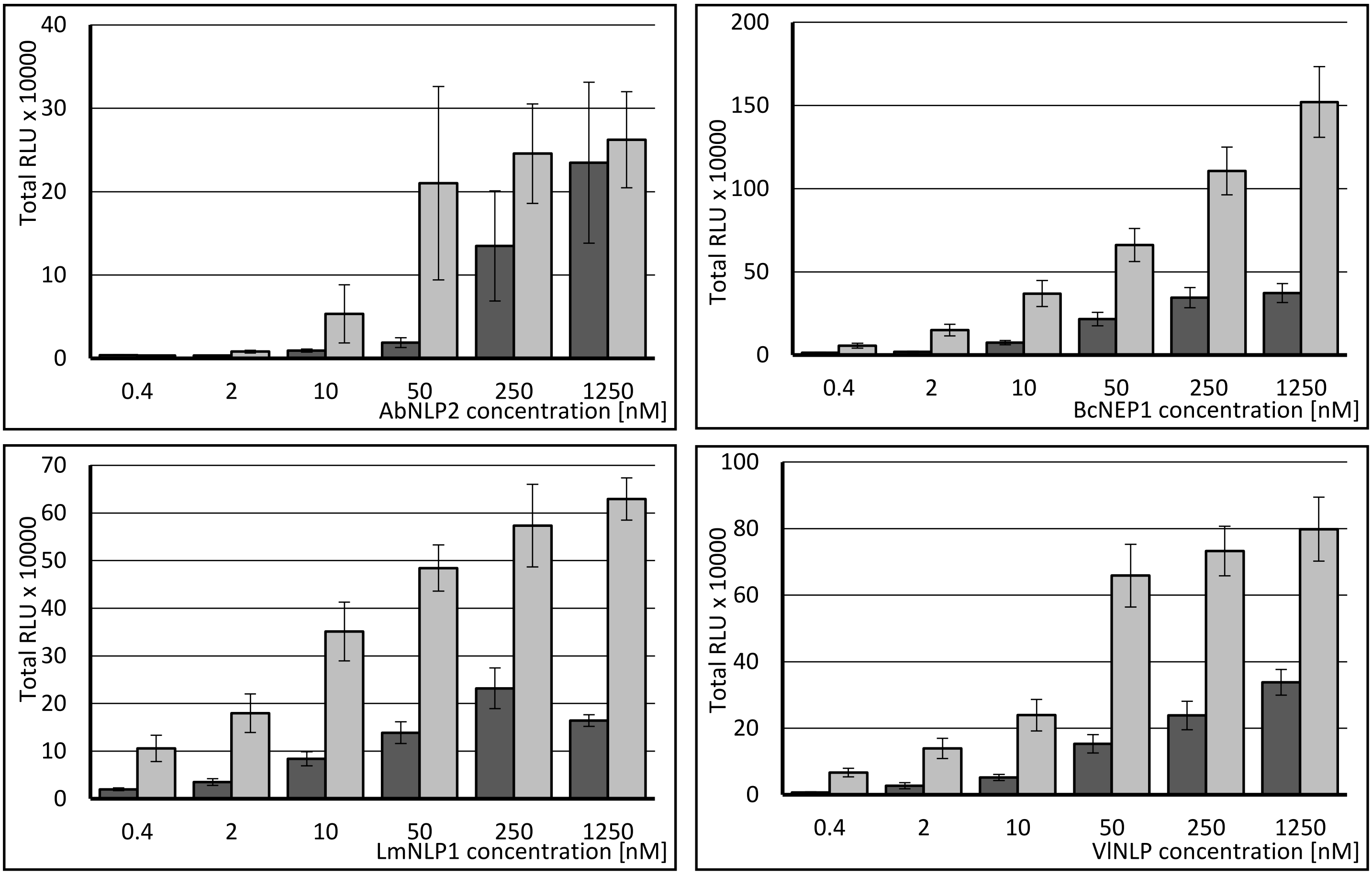

Figure S9. Effect of concentration of NLP peptides from Brassica pathogens pathogens *Alternaria brassicicola* (AbNLP2), *Botrytis cinerea* (BcNEP1), *Leptosphaeria maculans* (LmNLP1) and *Verticillium longisporum* (VlNLP1) on recognition in *B. napus* na1 (Ningyou1; dark grey) and na6 (NO2D-1952; light grey). Bars represent mean (+/- SEM) ROS response as relative light units (RLU) of at least 8 plants per concentration.
