# Supplementary material for: Necrosis and ethylene‐inducing‐like peptide patterns from crop pathogens induce differential responses within seven brassicaceous species
Source: Plant Pathol. 2022 Aug 5;71(9):2004–16. doi: 10.1111/ppa.13615 (PMC9804309; doi:10.1111/ppa.13615)
Supplement: Supplementary file 10 — Figure S10 [file PPA-71-2004-s003.pdf]

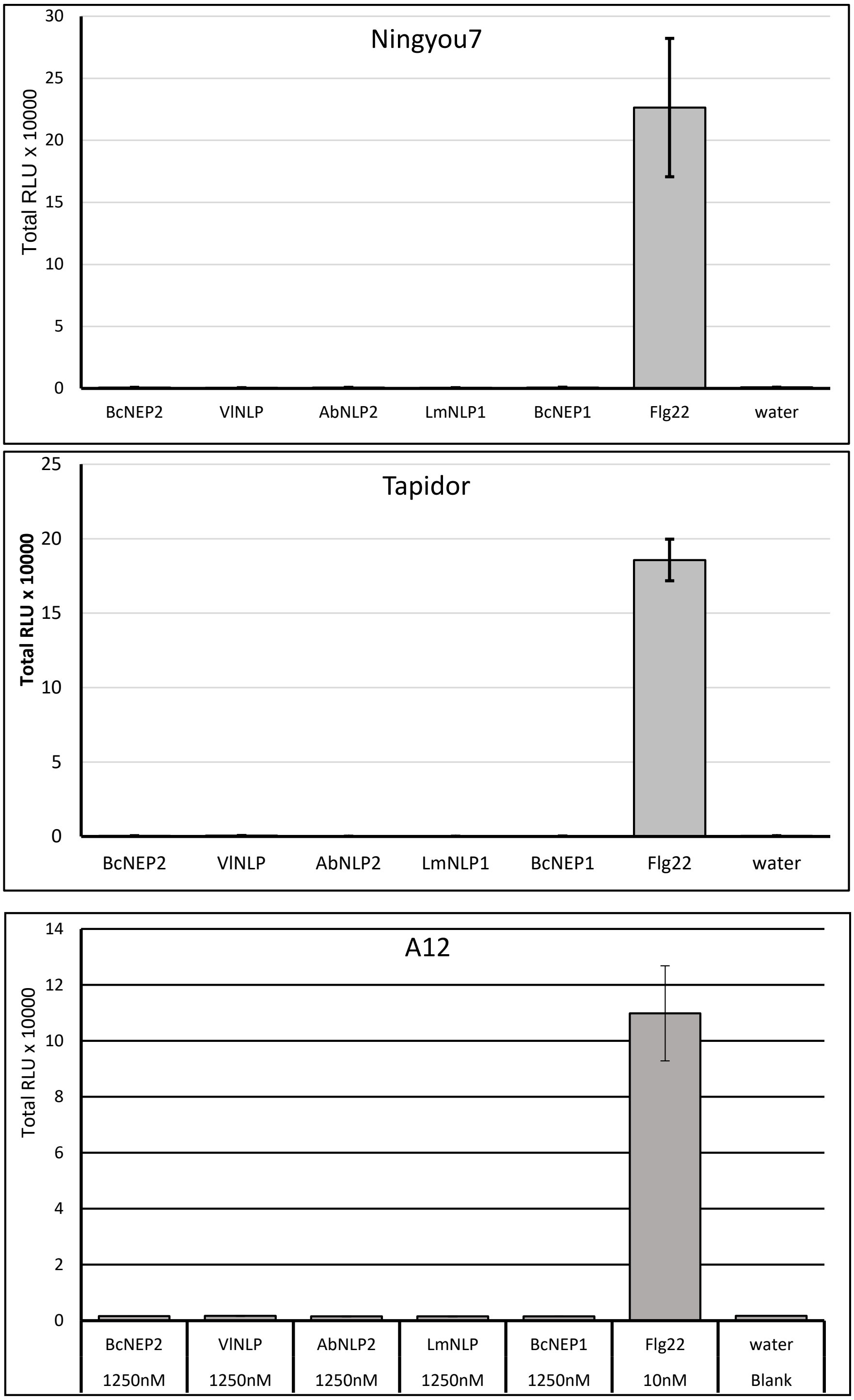

Figure S10. The MAMP motifs from NLPs of the Brassica pathogens *Alternaria brassicicola* (AbNLP2), *Botrytis cinerea* (BcNEP1 and BcNEP2), *Leptosphaeria maculans* (LmNLP1) and *Verticillium longisporum* (VlNLP1) are not recognised by plants that don't recognise BcNEP2. Leaf discs of (A) *B. napus* na8 (Ningyou7), (B) *B. napus* na3 (Tapidor) were challenged with 50 nM of each peptide and ROS-response recorded as total relative light units (RLU) over 40 min. Bars represent means (+/-SEM) of 3 individual experiments. (C) *B. oleracea* ol12 (A12DH) was challenged with 1250 nM of each NLP and 10 nM of flg22 and ROS-response recorded for 40 min. Bars represent means (+/-SEM) of 1 representative experiment.
