# Supplementary material for: Necrosis and ethylene‐inducing‐like peptide patterns from crop pathogens induce differential responses within seven brassicaceous species
Source: Plant Pathol. 2022 Aug 5;71(9):2004–16. doi: 10.1111/ppa.13615 (PMC9804309; doi:10.1111/ppa.13615)
Supplement: Supplementary file 11 — Figure S11 [file PPA-71-2004-s009.pdf]

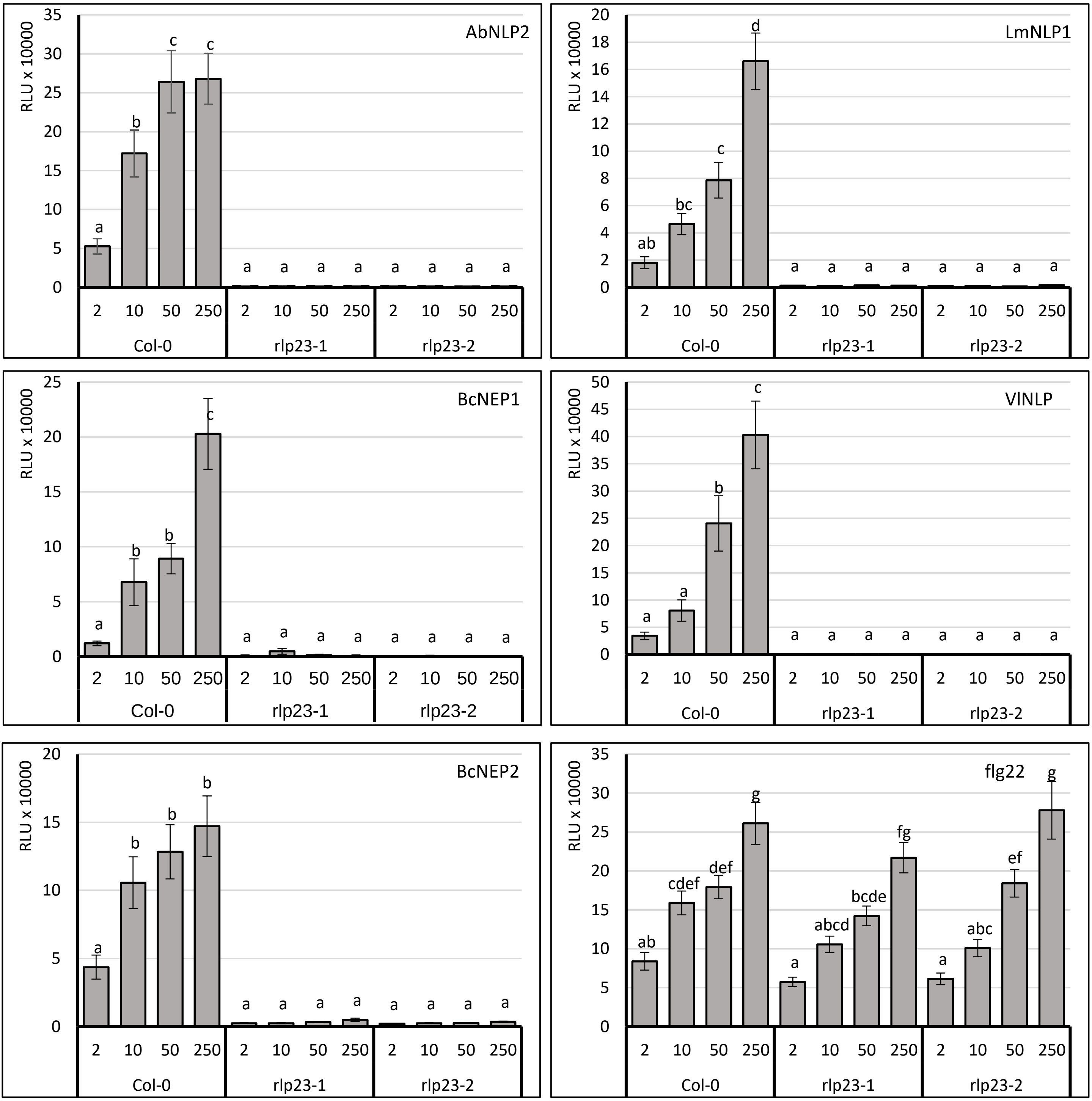

Figure S11. Effect of concentration of flg22 and NLP from crop-pathogens on recognition in Arabidopsis wild-type plants (Col-0) and 2 mutants of *Atrlp23*. The WT Col-0 and two independent mutant alleles *rlp23-1* and *rlp23-2* were challenged with 2, 10, 50 and 250 nM of each peptide and ROS-response recorded as total relative light units (RLU) over 40 min. Bars represent means (+/- SEM) of at least 3 individual experiments. Bars marked with different letters are significantly different ($P<0.05$) according to Fishers unprotected LSD in an ANOVA with unbalanced design.
