# Supplementary material for: Necrosis and ethylene‐inducing‐like peptide patterns from crop pathogens induce differential responses within seven brassicaceous species
Source: Plant Pathol. 2022 Aug 5;71(9):2004–16. doi: 10.1111/ppa.13615 (PMC9804309; doi:10.1111/ppa.13615)
Supplement: Supplementary file 12 — Figure S12 [file PPA-71-2004-s024.pdf]

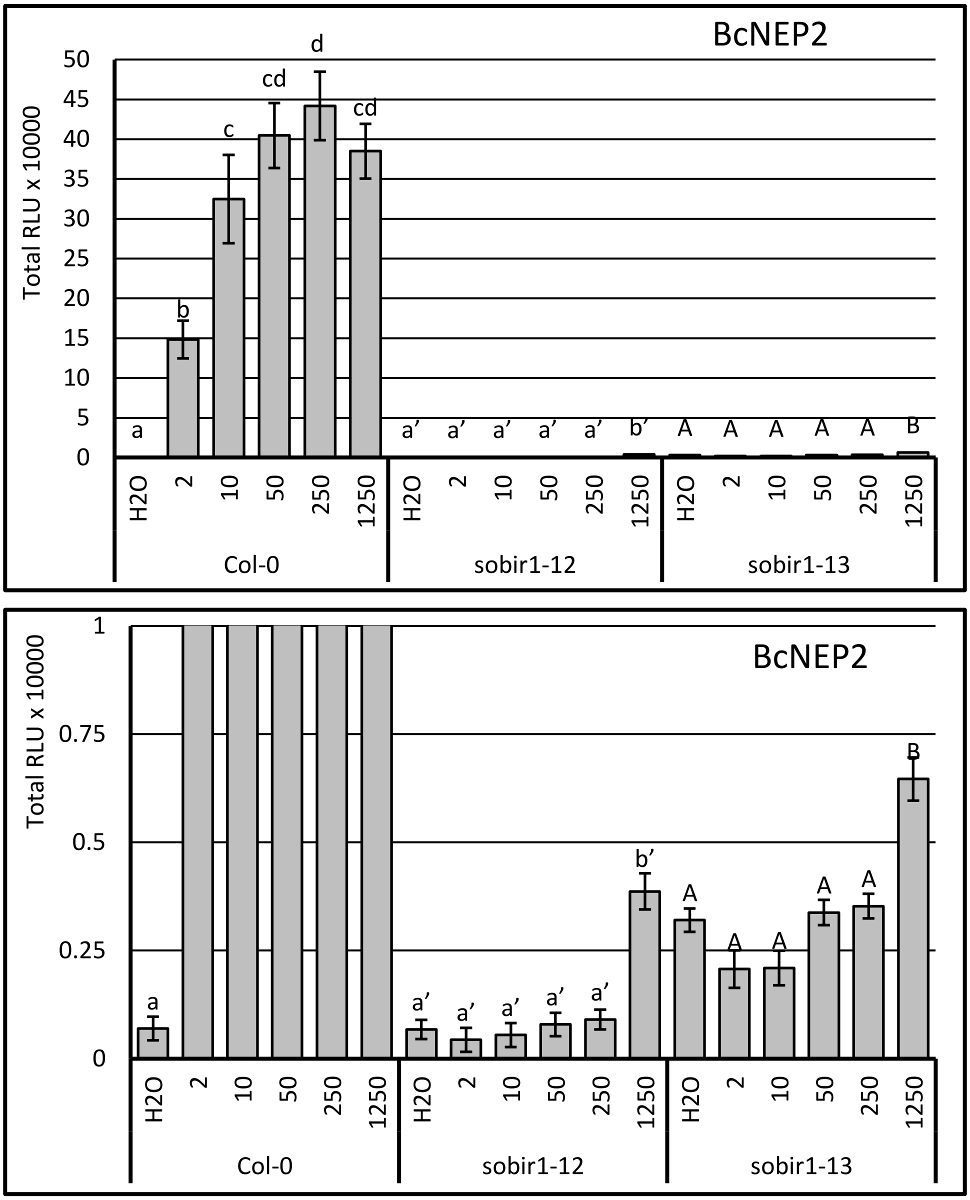

FigS12. Effect of concentration of the BcNEP2 peptide on recognition in Arabidopsis wild-type plants (Col-0) and 2 mutants of *Atsobir1*. The WT Col-0 and two independent mutant alleles *sobir1-12* and *sobir1-13* were challenged with water or 2, 10, 50, 250 and 1250 nM of BcNEP2 and ROS-response recorded as total relative light units (RLU) over 40 min. Bars represent means (+/- SEM) of 3 individual experiments with 8 leafdiscs per treatment each. Bars marked with different letters are significantly different ($P<0.05$) according to Fishers unprotected LSD in an ANOVA with unbalanced design, with statistical analysis performed separately per plant genotype.
