# Supplementary material for: Necrosis and ethylene‐inducing‐like peptide patterns from crop pathogens induce differential responses within seven brassicaceous species
Source: Plant Pathol. 2022 Aug 5;71(9):2004–16. doi: 10.1111/ppa.13615 (PMC9804309; doi:10.1111/ppa.13615)
Supplement: Supplementary file 13 — Figure S13 [file PPA-71-2004-s012.pdf]

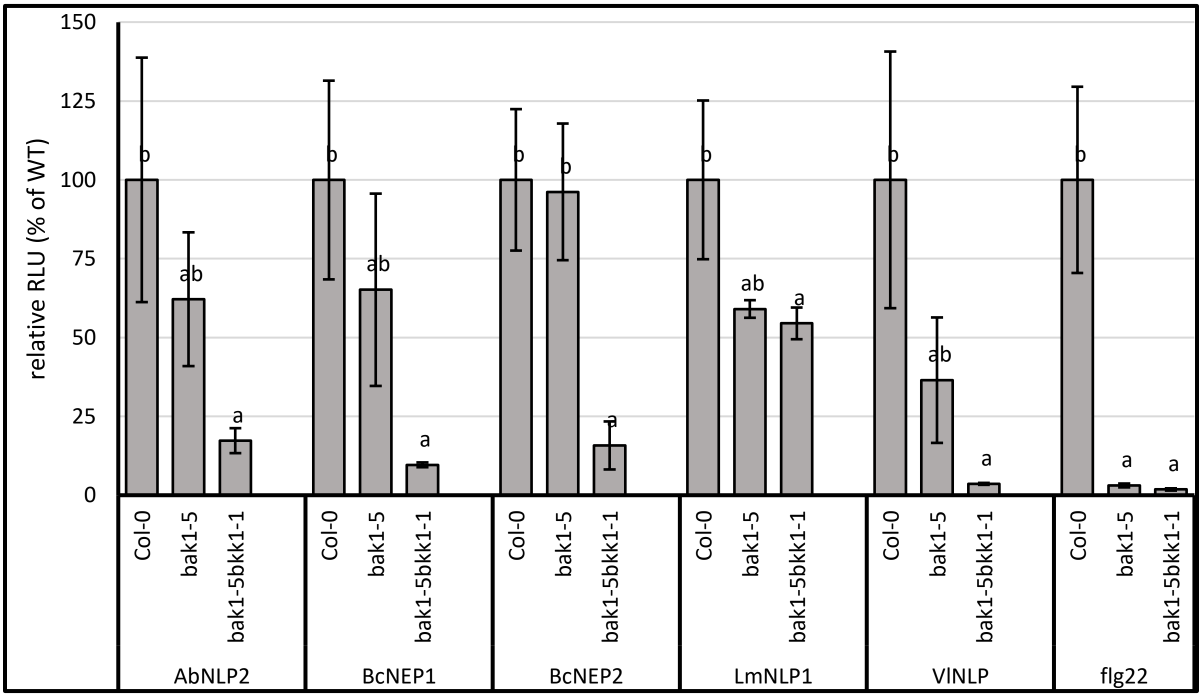

Figure S13
Role of SERKs in ROS-response to NLPs from crop pathogens. The wild-type Col-0 and mutants *bak1-5* and *bak1-5bkk1-1* were treated with 50 nM of each NLP peptide from crop-pathogens and flg22. Within each experiment the ROS response of each leafdisc (WT or mutant) was expressed as percentage of the average Col-0 response prior to statistical analysis. Bars represent means (+/-SEM) of at least 3 individual experiments with 8 leafdiscs each. Bars marked with different letters are significantly different ($P<0.05$) according to Fishers unprotected LSD in an ANOVA with unbalanced design.
