# Supplementary material for: Necrosis and ethylene‐inducing‐like peptide patterns from crop pathogens induce differential responses within seven brassicaceous species
Source: Plant Pathol. 2022 Aug 5;71(9):2004–16. doi: 10.1111/ppa.13615 (PMC9804309; doi:10.1111/ppa.13615)
Supplement: Supplementary file 14 — Figure S14 [file PPA-71-2004-s014.pdf]

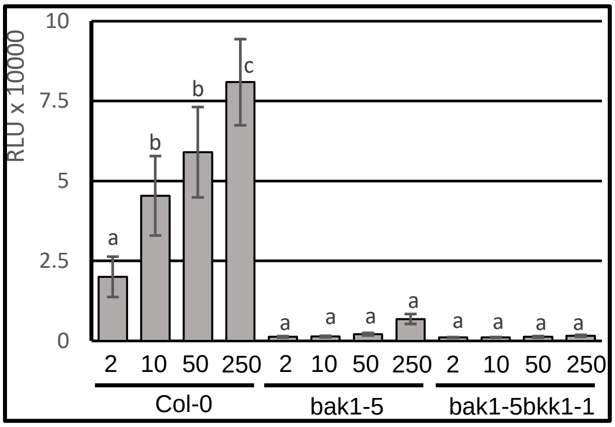

Figure S14. Effect of concentration of flg22 on recognition in Arabidopsis wild-type plants (Col-0) and SERK-mutants. The wild-type Col-0 and mutants *bak1-5* and *bak1-5bkk1-1* were challenged with 2, 10, 50 and 250 nM of flg22 and ROS-response recorded as total relative light units (RLU) for 40 min. Bars represent means (+/-SEM) of at least 3 individual experiments. Bars marked with different letters are significantly different (P<0.05) according to Fishers unprotected LSD in an ANOVA with unbalanced design.
