# Supplementary material for: Necrosis and ethylene‐inducing‐like peptide patterns from crop pathogens induce differential responses within seven brassicaceous species
Source: Plant Pathol. 2022 Aug 5;71(9):2004–16. doi: 10.1111/ppa.13615 (PMC9804309; doi:10.1111/ppa.13615)
Supplement: Supplementary file 15 — Figure S15 [file PPA-71-2004-s019.pdf]

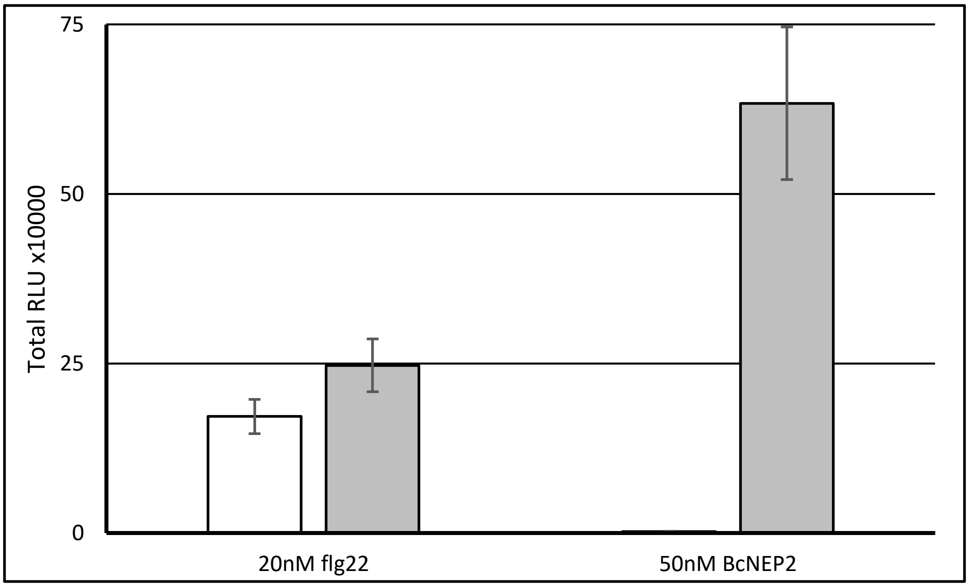

Figure S15. PAMP response in *B. napus* cultivars with a publicly available genome sequence. Total ROS response of Darmor (white bars) and Zhongshuang11 (grey bars) treated for 40 min with 20 nM flg22 or 50 nM BcNEP2 measured as relative light unites (RLU). Bars display the mean (+/- SEM) of 8 plants from 2 different seed packages with 2 leafdiscs each.
