# Supplementary material for: Necrosis and ethylene‐inducing‐like peptide patterns from crop pathogens induce differential responses within seven brassicaceous species
Source: Plant Pathol. 2022 Aug 5;71(9):2004–16. doi: 10.1111/ppa.13615 (PMC9804309; doi:10.1111/ppa.13615)
Supplement: Supplementary file 16 — Figure S16 [file PPA-71-2004-s008.pdf]

SOBIR

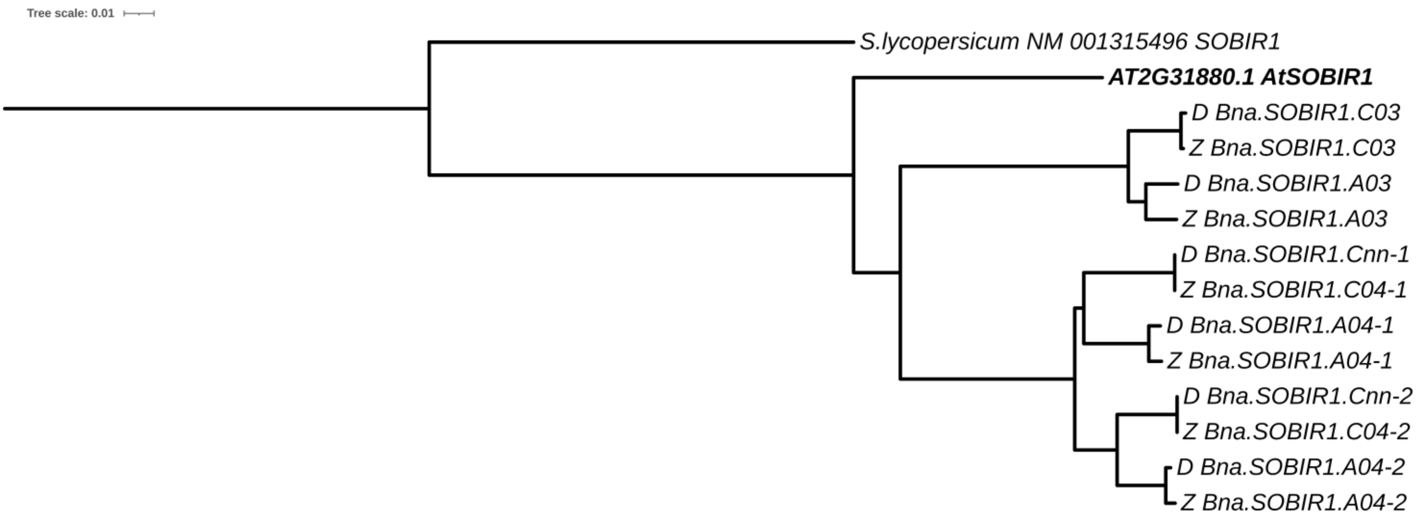

Figure S16
Phylogenetic relationship of SOBIR genes from *Brassica napus (Bna)s* and Arabidopsis (*At*).
Phylogenetic relationship of *AtSOBIR1* orthologs on the Darmor-*bzh* (D *Bna*) and Zhongshuang11 (Z *Bna*) genome. The alignment and tree were generated using ClustalW and the visualized via iTOL. The phylogenetic tree was calculated via the neighbour-joining method on genomic DNA. *AtSOBIR1* is labelled in bold. The tomato gene *SlSOBIR1* from *Solanum lycopersicum* is used as outgroup.
