# Supplementary material for: Necrosis and ethylene‐inducing‐like peptide patterns from crop pathogens induce differential responses within seven brassicaceous species
Source: Plant Pathol. 2022 Aug 5;71(9):2004–16. doi: 10.1111/ppa.13615 (PMC9804309; doi:10.1111/ppa.13615)
Supplement: Supplementary file 17 — Figure S17 [file PPA-71-2004-s005.pdf]

SERKs

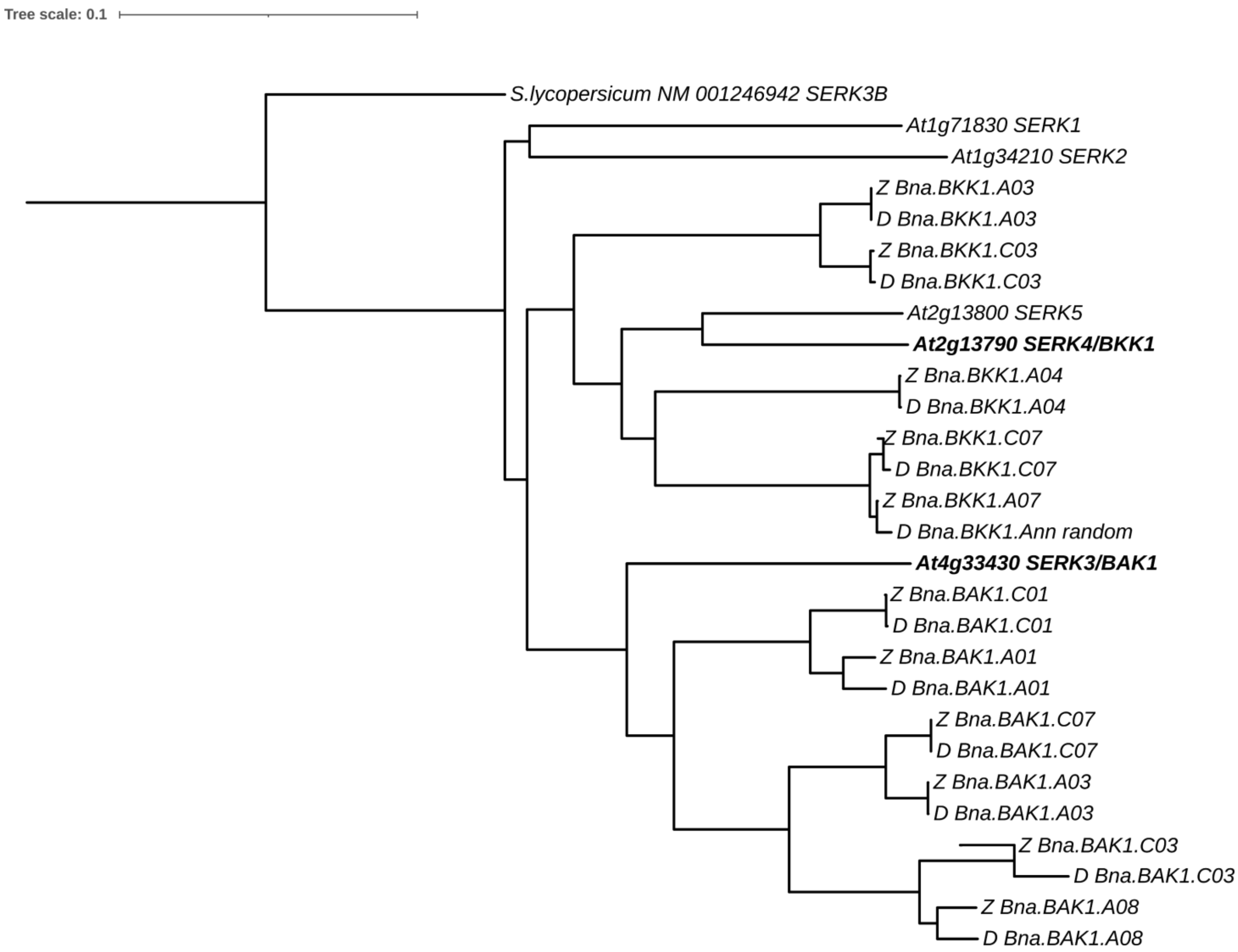

Figure S17
Phylogenetic relationship of SERK genes from *Brassica Napus (Bna)* and Arabidopsis(*At*). Phylogenetic relationship of *AtBAK1* and *AtBKK1* orthologs on the Darmor-*bzh* (D Bna) and Zhongshuang11 (Z Bna) genome. The alignment and tree were generated using Clustal Omega and the visualized via iTOL. The phylogenetic trees were calculated via the neighbour-joining method on genomic DNA corresponding to the positions from start to stop codon in the most related Arabidopsis gene. *AtBAK1* and *AtBKK1* are labelled in bold. The tomato gene *SlSERK3B* from *S. lycopersicum* is used as outgroup.
