# Supplementary material for: Necrosis and ethylene‐inducing‐like peptide patterns from crop pathogens induce differential responses within seven brassicaceous species
Source: Plant Pathol. 2022 Aug 5;71(9):2004–16. doi: 10.1111/ppa.13615 (PMC9804309; doi:10.1111/ppa.13615)
Supplement: Supplementary file 19 — Figure S19 [file PPA-71-2004-s022.pdf]

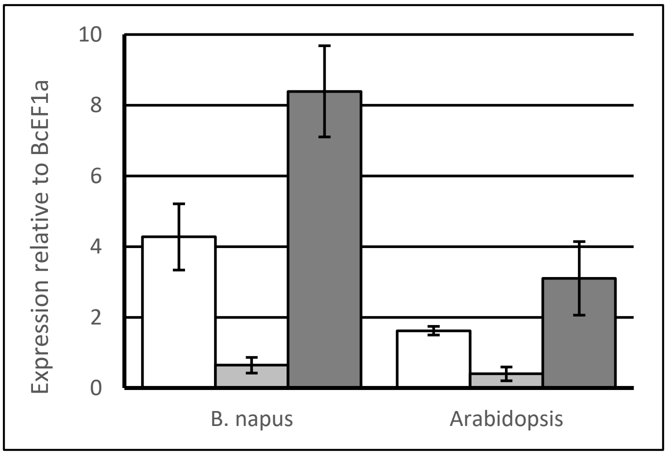

Figure S19

Bcnep1 and *Bcnep2* are expressed *in planta*. 24 hours after spray-inoculation with B05.10 on *A. thaliana* Col-0 or *B. napus* cv. Temple both *Bcnep1* (light grey) and *Bcnep2* (dark grey) are expressed to a similar extend as the known *Botrytis cinerea* elicitor *Bcxyn11A* (white). Bars represent means (+/-SEM) of 3 biological samples.
