# Supplementary material for: Necrosis and ethylene‐inducing‐like peptide patterns from crop pathogens induce differential responses within seven brassicaceous species
Source: Plant Pathol. 2022 Aug 5;71(9):2004–16. doi: 10.1111/ppa.13615 (PMC9804309; doi:10.1111/ppa.13615)
Supplement: Supplementary file 23 — Table S4 [file PPA-71-2004-s015.pdf]

Table S4

Annotation and effect of predicted variants found in Darmor of homologs with corresponding locations shown on the Darmor-*bzh* genome.

**Gene-ID**	**Annotated Gene**	**Chromosome**	**Position**	**Reference**	**Alternative**	**Type**	**Quality**	**Effect**	**Effect Impact**
D_Bna.RLP23.C04-8	BnaC04g43250D	chrC04	43602261	T	G	snp	735.818	missense_variant	MODERATE
D_Bna.RLP23.C04-8	BnaC04g43250D	chrC04	43602327	A	C	snp	820.068	missense_variant	MODERATE
D_Bna.RLP23.C04-9	BnaC04g43260D	chrC04	43612317	G	T	snp	1059.99	intron_variant	MODIFIER
D_Bna.BKK1.A03	BnaA03g38480D	chrA03	19129077	TAAAAAAAAAT	TAAAAAAAAAAT	insertion	628.93	intron_variant	MODIFIER
