# Supplementary material for: Necrosis and ethylene‐inducing‐like peptide patterns from crop pathogens induce differential responses within seven brassicaceous species
Source: Plant Pathol. 2022 Aug 5;71(9):2004–16. doi: 10.1111/ppa.13615 (PMC9804309; doi:10.1111/ppa.13615)
Supplement: Supplementary file 18 — Figure S18 [file PPA-71-2004-s020.pdf]

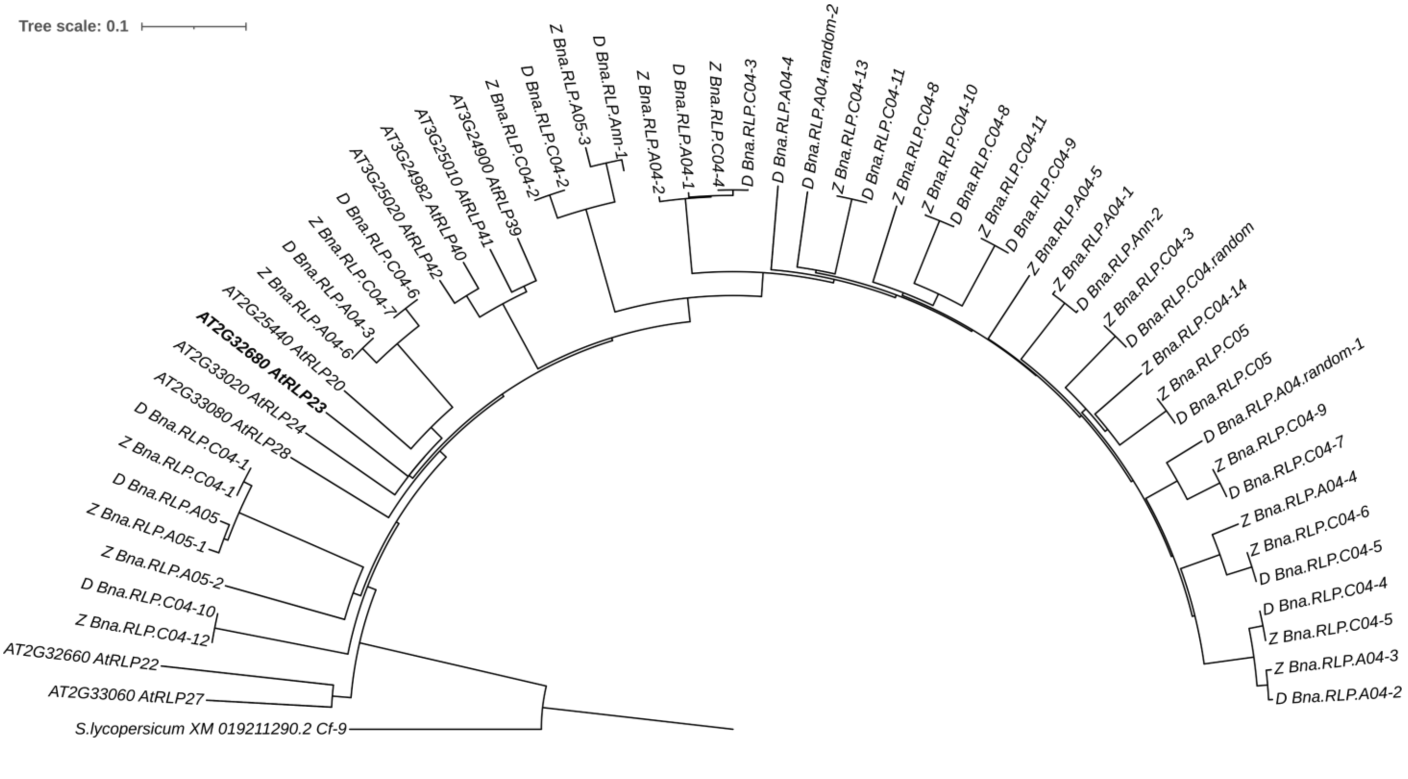

Figure S18

Phylogenetic relationship of Arabidopsis and *B. napus* genes with >60% homology to *AtRLP23*. Phylogenetic relationship of *AtRLP23* orthologs on the Darmor-*bzh* (D *Bna*) and Zhongshuang11 (Z *Bna*) genome. The alignment and tree were generated using ClustalW and the visualized via iTOL. The phylogenetic tree was calculated with the neighbour-joining method on genomic DNA corresponding to the positions from start to stop codon in the most related Arabidopsis gene. *AtRLP23* is labelled in bold. The tomato gene *SlCf-9* from *S. lycopersicum* is used as outgroup.
